# On a Generalization of the Jensen–Shannon Divergence and the Jensen–Shannon Centroid

**DOI:** 10.3390/e22020221

**Published:** 2020-02-16

**Authors:** Frank Nielsen

**Affiliations:** Sony Computer Science Laboratories, Tokyo 141-0022, Japan; Frank.Nielsen@acm.org

**Keywords:** Bregman divergence, *f*-divergence, Jensen–Bregman divergence, Jensen diversity, Jensen–Shannon divergence, capacitory discrimination, Jensen–Shannon centroid, mixture family, information geometry, difference of convex (DC) programming

## Abstract

The Jensen–Shannon divergence is a renown bounded symmetrization of the Kullback–Leibler divergence which does not require probability densities to have matching supports. In this paper, we introduce a vector-skew generalization of the scalar α-Jensen–Bregman divergences and derive thereof the vector-skew α-Jensen–Shannon divergences. We prove that the vector-skew α-Jensen–Shannon divergences are *f*-divergences and study the properties of these novel divergences. Finally, we report an iterative algorithm to numerically compute the Jensen–Shannon-type centroids for a set of probability densities belonging to a mixture family: This includes the case of the Jensen–Shannon centroid of a set of categorical distributions or normalized histograms.

## 1. Introduction

Let (X,F,μ) be a measure space [1] where X denotes the sample space, F the σ-algebra of measurable events, and μ a positive measure; for example, the measure space defined by the Lebesgue measure $\mu_{L}$ with Borel σ-algebra $B(R^{d})$ for $X=R^{d}$ or the measure space defined by the counting measure $\mu_{c}$ with the power set σ-algebra $2^{X}$ on a finite alphabet X. Denote by $L_{1}\left(X,F,\mu \right)$ the Lebesgue space of measurable functions, $P_{1}$ the subspace of *positive* integrable functions *f* such that $\int_{X}f\left(x\right)d\mu \left(x\right)=1$ and f(x)>0 for all x∈X, and ${\bar{P}}_{1}$ the subspace of *non-negative* integrable functions *f* such that $\int_{X}f\left(x\right)d\mu \left(x\right)=1$ and f(x)≥0 for all x∈X.

We refer to the book of Deza and Deza [2] and the survey of Basseville [3] for an introduction to the many types of statistical divergences met in information sciences and their justifications. The *Kullback–Leibler Divergence* (KLD) $\mathrm{KL}:P_{1}\times P_{1}\to \left[0,\infty \right]$ is an oriented statistical distance (commonly called the relative entropy in information theory [4]) defined between two densities *p* and *q* (i.e., the Radon–Nikodym densities of μ-absolutely continuous probability measures *P* and *Q*) by

(1) $$\mathrm{KL}\left(p:q\right):=\int p\log\frac{p}{q}d\mu .$$

Although KL(p:q)≥0 with equality iff. p=qμ-a. e. (Gibb’s inequality [4]), the KLD may diverge to infinity depending on the underlying densities. Since the KLD is asymmetric, several symmetrizations [5] have been proposed in the literature.

A well-grounded symmetrization of the KLD is the *Jensen–Shannon Divergence* [6] (JSD), also called *capacitory discrimination* in the literature (e.g., see [7]):

(2) $$\begin{array}{ccc} \mathrm{JS}(p,q) & := & \frac{1}{2}\left(\mathrm{KL}\left(p:\frac{p+q}{2}\right)+\mathrm{KL}\left(q:\frac{p+q}{2}\right)\right), \end{array}$$

(3) $$\begin{array}{cc} = & \frac{1}{2}\int \left(p\log\frac{2p}{p+q}+q\log\frac{2q}{p+q}\right)d\mu =\mathrm{JS}\left(q,p\right). \end{array}$$

The Jensen–Shannon divergence can be interpreted as the *total KL divergence to the average distribution* $\frac{p+q}{2}$. The Jensen–Shannon divergence was historically implicitly introduced in [8] (Equation (19)) to calculate distances between random graphs. A nice feature of the Jensen–Shannon divergence is that this divergence can be applied to densities with *arbitrary* support (i.e., $p,q\in {\bar{P}}_{1}$ with the convention that 0log0=0 and $\log\frac{0}{0}=0$); moreover, the JSD is *always* upper bounded by log2. Let $X_{p}=\mathrm{supp}\left(p\right)$ and $X_{q}=\mathrm{supp}\left(q\right)$ denote the supports of the densities *p* and *q*, respectively, where supp(p):={x∈X:p(x)>0}. The JSD saturates to log2 whenever the supports $X_{p}$ and $X_{p}$ are disjoints. We can rewrite the JSD as

(4) $$\mathrm{JS}\left(p,q\right)=h\left(\frac{p+q}{2}\right)-\frac{h(p)+h(q)}{2},$$

where h(p)=−∫plogpdμ denotes Shannon’s entropy. Thus, the JSD can also be interpreted as the *entropy of the average distribution minus the average of the entropies*.

The square root of the JSD is a metric [9] satisfying the triangle inequality, but the square root of the JD is not a metric (nor any positive power of the Jeffreys divergence, see [10]). In fact, the JSD can be interpreted as a Hilbert metric distance, meaning that there exists some isometric embedding of $\left(X,\sqrt{\mathrm{JS}}\right)$ into a Hilbert space [11,12]. Other principled symmetrizations of the KLD have been proposed in the literature: For example, Naghshvar et al. [13] proposed the *extrinsic Jensen–Shannon divergence* and demonstrated its use for variable-length coding over a discrete memoryless channel (DMC).

Another symmetrization of the KLD sometimes met in the literature [14,15,16] is the *Jeffreys divergence* [17,18] (JD) defined by

(5) $$J\left(p,q\right):=\mathrm{KL}\left(p:q\right)+\mathrm{KL}\left(q:p\right)=\int \left(p-q\right)\log\frac{p}{q}d\mu =J\left(q,p\right).$$

However, we point out that this Jeffreys divergence lacks sound information-theoretical justifications.

For two positive but not necessarily normalized densities $\tilde{p}$ and $\tilde{q}$, we define the *extended Kullback–Leibler divergence* as follows:

(6) $$\begin{array}{ccc} {\mathrm{KL}}^{+}\left(\tilde{p}:\tilde{q}\right) & := & \mathrm{KL}\left(\tilde{p}:\tilde{q}\right)+\int \tilde{q}d\mu -\int \tilde{p}d\mu , \end{array}$$

(7) $$\begin{array}{cc} = & \int \left(\tilde{p}\log\frac{\tilde{p}}{\tilde{q}}+\tilde{q}-\tilde{p}\right)d\mu . \end{array}$$

The Jensen–Shannon divergence and the Jeffreys divergence can both be extended to positive (unnormalized) densities without changing their formula expressions:

(8) $$\begin{array}{ccc} {\mathrm{JS}}^{+}\left(\tilde{p},\tilde{q}\right) & := & \frac{1}{2}\left({\mathrm{KL}}^{+}\left(\tilde{p}:\frac{\tilde{p}+\tilde{q}}{2}\right)+{\mathrm{KL}}^{+}\left(\tilde{q}:\frac{\tilde{p}+\tilde{q}}{2}\right)\right), \end{array}$$

(9) $$\begin{array}{cc} = & \frac{1}{2}\left(\mathrm{KL}\left(\tilde{p}:\frac{\tilde{p}+\tilde{q}}{2}\right)+\mathrm{KL}\left(\tilde{q}:\frac{\tilde{p}+\tilde{q}}{2}\right)\right)=\mathrm{JS}\left(\tilde{p},\tilde{q}\right), \end{array}$$

(10) $$\begin{array}{ccc} J^{+}\left(\tilde{p},\tilde{q}\right) & := & {\mathrm{KL}}^{+}\left(\tilde{p}:\tilde{q}\right)+{\mathrm{KL}}^{+}\left(\tilde{p}:\tilde{q}\right)=\int \left(\tilde{p}-\tilde{q}\right)\log\frac{\tilde{p}}{\tilde{q}}d\mu =J\left(\tilde{p},\tilde{q}\right). \end{array}$$

However, the extended ${\mathrm{JS}}^{+}$ divergence is upper-bounded by $\left(\frac{1}{2}\log2\right)\left(\int \left(\tilde{p}+\tilde{q}\right)d\mu \right)=\frac{1}{2}\left(\mu \left(p\right)+\mu \left(q\right)\right)\log2$ instead of log2 for normalized densities (i.e., when μ(p)+μ(q)=2).

Let ${\left(pq\right)}_{\alpha }\left(x\right):=\left(1-\alpha \right)p\left(x\right)+\alpha q\left(x\right)$ denote the statistical weighted mixture with component densities *p* and *q* for α∈[0,1]. The asymmetric α-skew Jensen–Shannon divergence can be defined for a scalar parameter α∈(0,1) by considering the weighted mixture ${\left(pq\right)}_{\alpha }$ as follows:

(11) $$\begin{array}{ccc} {\mathrm{JS}}_{a}^{\alpha }\left(p:q\right) & := & \left(1-\alpha \right)\mathrm{KL}\left(p:{\left(pq\right)}_{\alpha }\right)+\alpha \mathrm{KL}\left(q:{\left(pq\right)}_{\alpha }\right), \end{array}$$

(12) $$\begin{array}{cc} = & \left(1-\alpha \right)\int p\log\frac{p}{{\left(pq\right)}_{\alpha }}d\mu +\alpha \int q\log\frac{q}{{\left(pq\right)}_{\alpha }}d\mu . \end{array}$$

Let us introduce the *α-skew K-divergence* [6,19] $K_{\alpha }\left(p:q\right)$ by:

(13) $$K_{\alpha }\left(p:q\right):=\mathrm{KL}\left(p:(1-\alpha )p+\alpha q\right)=\mathrm{KL}\left(p:{\left(pq\right)}_{\alpha }\right).$$

Then, both the Jensen–Shannon divergence and the Jeffreys divergence can be rewritten [20] using $K_{\alpha }$ as follows:

(14) $$\begin{array}{ccc} \mathrm{JS}\left(p,q\right) & = & \frac{1}{2}\left(K_{\frac{1}{2}}\left(p:q\right)+K_{\frac{1}{2}}\left(q:p\right)\right), \end{array}$$

(15) $$\begin{array}{ccc} J\left(p,q\right) & = & K_{1}\left(p:q\right)+K_{1}\left(q:p\right), \end{array}$$

since ${\left(pq\right)}_{1}=q$, $\mathrm{KL}\left(p:q\right)=K_{1}\left(p:q\right)$ and ${\left(pq\right)}_{\frac{1}{2}}={\left(qp\right)}_{\frac{1}{2}}$.

We can thus define the *symmetric* *α-skew Jensen–Shannon divergence* [20] for α∈(0,1) as follows:

(16) $$\begin{array}{ccc} {\mathrm{JS}}^{\alpha }\left(p,q\right) & := & \frac{1}{2}K_{\alpha }\left(p:q\right)+\frac{1}{2}K_{\alpha }\left(q:p\right)={\mathrm{JS}}^{\alpha }\left(q,p\right). \end{array}$$

The ordinary Jensen–Shannon divergence is recovered for $\alpha =\frac{1}{2}$.

In general, skewing divergences (e.g., using the divergence $K_{\alpha }$ instead of the KLD) have been experimentally shown to perform better in applications like in some natural language processing (NLP) tasks [21].

The *α-Jensen–Shannon divergences* are Csiszár *f*-divergences [22,23,24]. An *f*-divergence is defined for a convex function *f*, strictly convex at 1, and satisfies f(1)=0 as:

(17) $$I_{f}\left(p:q\right)=\int q\left(x\right)f\left(\frac{p(x)}{q(x)}\right)dx\geq f\left(1\right)=0.$$

We can always symmetrize *f*-divergences by taking the *conjugate* convex function $f^{*}\left(x\right)=xf\left(\frac{1}{x}\right)$ (related to the perspective function): $I_{f+f^{*}}\left(p,q\right)$ is a symmetric divergence. The *f*-divergences are convex statistical distances which are provably the only separable invariant divergences in information geometry [25], except for binary alphabets X (see [26]).

The Jeffreys divergence is an *f*-divergence for the generator f(x)=(x−1)logx, and the α-Jensen–Shannon divergences are *f*-divergences for the generator family $f_{\alpha }\left(x\right)=-\log\left(\left(1-\alpha \right)+\alpha x\right)-x\log\left(\left(1-\alpha \right)+\frac{\alpha }{x}\right)$. The *f*-divergences are upper-bounded by $f\left(0\right)+f^{*}\left(0\right)$. Thus, the *f*-divergences are finite when $f\left(0\right)+f^{*}\left(0\right)<\infty$.

The main contributions of this paper are summarized as follows:

- First, we generalize the Jensen–Bregman divergence by skewing a weighted separable Jensen–Bregman divergence with a *k*-dimensional *vector* $\alpha \in {\left[0,1\right]}^{k}$ in Section 2. This yields a generalization of the symmetric skew α-Jensen–Shannon divergences to a vector-skew parameter. This extension retains the key properties for being upper-bounded and for application to densities with potentially different supports. The proposed generalization also allows one to grasp a better understanding of the “mechanism” of the Jensen–Shannon divergence itself. We also show how to directly obtain the weighted vector-skew Jensen–Shannon divergence from the decomposition of the KLD as the difference of the cross-entropy minus the entropy (i.e., KLD as the relative entropy).
- Second, we prove that weighted vector-skew Jensen–Shannon divergences are *f*-divergences (Theorem 1), and show how to build families of symmetric Jensen–Shannon-type divergences which can be controlled by a vector of parameters in Section 2.3, generalizing the work of [20] from scalar skewing to vector skewing. This may prove useful in applications by providing additional tuning parameters (which can be set, for example, by using cross-validation techniques).
- Third, we consider the calculation of the *Jensen–Shannon centroids* in Section 3 for densities belonging to mixture families. Mixture families include the family of categorical distributions and the family of statistical mixtures sharing the same prescribed components. Mixture families are well-studied manifolds in information geometry [25]. We show how to compute the Jensen–Shannon centroid using a concave–convex numerical iterative optimization procedure [27]. The experimental results graphically compare the Jeffreys centroid with the Jensen–Shannon centroid for grey-valued image histograms.

## 2. Extending the Jensen–Shannon Divergence

### 2.1. Vector-Skew Jensen–Bregman Divergences and Jensen Diversities

Recall our notational shortcut: ${\left(ab\right)}_{\alpha }:=\left(1-\alpha \right)a+\alpha b$. For a *k*-dimensional vector $\alpha \in {\left[0,1\right]}^{k}$, a weight vector *w* belonging to the (k−1)-dimensional open simplex $\Delta_{k}$, and a scalar γ∈(0,1), let us define the following vector *skew α-Jensen–Bregman divergence* (α-JBD) following [28]:

(18) $${\mathrm{JB}}_{F}^{\alpha ,\gamma ,w}\left(\theta_{1}:\theta_{2}\right):=\sum_{i=1}^{k}w_{i}B_{F}\left({\left(\theta_{1}\theta_{2}\right)}_{\alpha_{i}}:{\left(\theta_{1}\theta_{2}\right)}_{\gamma }\right)\geq 0,$$

where $B_{F}$ is the *Bregman divergence* [29] induced by a strictly convex and smooth generator *F*:

(19) $$B_{F}\left(\theta_{1}:\theta_{2}\right):=F\left(\theta_{1}\right)-F\left(\theta_{2}\right)-\langle \theta_{1}-\theta_{2},\nabla F\left(\theta_{2}\right)\rangle ,$$

with 〈·,·〉 denoting the Euclidean inner product $\langle x,y\rangle =x^{⊤}y$ (dot product). Expanding the Bregman divergence formulas in the expression of the α-JBD and using the fact that

(20) $${\left(\theta_{1}\theta_{2}\right)}_{\alpha_{i}}-{\left(\theta_{1}\theta_{2}\right)}_{\gamma }=\left(\gamma -\alpha_{i}\right)\left(\theta_{1}-\theta_{2}\right),$$

we get the following expression:

(21) $${\mathrm{JB}}_{F}^{\alpha ,\gamma ,w}\left(\theta_{1}:\theta_{2}\right)=\left(\sum_{i=1}^{k}w_{i}F\left({\left(\theta_{1}\theta_{2}\right)}_{\alpha_{i}}\right)\right)-F\left({\left(\theta_{1}\theta_{2}\right)}_{\gamma }\right)-\langle \sum_{i=1}^{k}w_{i}\left(\gamma -\alpha_{i}\right)\left(\theta_{1}-\theta_{2}\right),\nabla F\left({\left(\theta_{1}\theta_{2}\right)}_{\gamma }\right)\rangle .$$

The inner product term of Equation (21) vanishes when

(22) $$\gamma =\sum_{i=1}^{k}w_{i}\alpha_{i}:=\bar{\alpha }.$$

Thus, when $\gamma =\bar{\alpha }$ (assuming at least two distinct components in α so that γ∈(0,1)), we get the simplified formula for the vector-skew α-JBD:

(23) $$\begin{array}{c} {\mathrm{JB}}_{F}^{\alpha ,w}\left(\theta_{1}:\theta_{2}\right)=\left(\sum_{i=1}^{k}w_{i}F\left({\left(\theta_{1}\theta_{2}\right)}_{\alpha_{i}}\right)\right)-F\left({\left(\theta_{1}\theta_{2}\right)}_{\bar{\alpha }}\right). \end{array}$$

This vector-skew Jensen–Bregman divergence is always finite and amounts to a *Jensen diversity* [30] $J_{F}$ induced by Jensen’s inequality gap:

(24) $${\mathrm{JB}}_{F}^{\alpha ,w}\left(\theta_{1}:\theta_{2}\right)=J_{F}\left({\left(\theta_{1}\theta_{2}\right)}_{\alpha_{1}},\ldots ,{\left(\theta_{1}\theta_{2}\right)}_{\alpha_{k}};w_{1},\ldots ,w_{k}\right):=\sum_{i=1}^{k}w_{i}F\left({\left(\theta_{1}\theta_{2}\right)}_{\alpha_{i}}\right)-F\left({\left(\theta_{1}\theta_{2}\right)}_{\bar{\alpha }}\right)\geq 0.$$

The Jensen diversity is a quantity which arises as a generalization of the cluster variance when clustering with Bregman divergences instead of the ordinary squared Euclidean distance; see [29,30] for details. In the context of Bregman clustering, the Jensen diversity has been called the *Bregman information* [29] and motivated by rate distortion theory: Bregman information measures the minimum expected loss when encoding a set of points using a single point when the loss is measured using a Bregman divergence. In general, a *k*-point measure is called a diversity measure (for k>2), while a distance/divergence is the special case of a 2-point measure.

Conversely, in 1D, we may start from Jensen’s inequality for a strictly convex function *F*:

(25) $$\sum_{i=1}^{k}w_{i}F\left(\theta_{i}\right)\geq F\left(\sum_{i=1}^{k}w_{i}\theta_{i}\right).$$

Let us notationally write [k]:={1,…,k}, and define $\theta_{m}:=\min_{i\in [k]}{\left\{\theta_{i}\right\}}_{i}$ and $\theta_{M}:=\max_{i\in [k]}{\left\{\theta_{i}\right\}}_{i}>\theta_{m}$ (i.e., assuming at least two distinct values). We have the barycenter $\bar{\theta }=\sum_{i}w_{i}\theta_{i}=:{\left(\theta_{m}\theta_{M}\right)}_{\gamma }$ which can be interpreted as the linear interpolation of the extremal values for some γ∈(0,1). Let us write $\theta_{i}={\left(\theta_{m}\theta_{M}\right)}_{\alpha_{i}}$ for i∈[k] and proper values of the $\alpha_{i}$s. Then, it comes that

(26) $$\begin{array}{ccc} \bar{\theta } & = & \sum_{i}w_{i}\theta_{i}, \end{array}$$

(27) $$\begin{array}{cc} = & \sum_{i}w_{i}{\left(\theta_{m}\theta_{M}\right)}_{\alpha_{i}}, \end{array}$$

(28) $$\begin{array}{cc} = & \sum_{i}w_{i}\left(\left(1-\alpha_{i}\right)\theta_{m}+\alpha_{i}\theta_{M}\right), \end{array}$$

(29) $$\begin{array}{cc} = & \left(1-\sum_{i}w_{i}\alpha_{i}\right)\theta_{m}+\sum_{i}\alpha_{i}w_{i}\theta_{M}, \end{array}$$

(30) $$\begin{array}{cc} = & {\left(\theta_{m}\theta_{M}\right)}_{\sum_{i}w_{i}\alpha_{i}}={\left(\theta_{m}\theta_{M}\right)}_{\gamma }, \end{array}$$

so that $\gamma =\sum_{i}w_{i}\alpha_{i}=\bar{\alpha }$.

### 2.2. Vector-Skew Jensen–Shannon Divergences

Let f(x)=xlogx−x be a strictly smooth convex function on (0,∞). Then, the Bregman divergence induced by this univariate generator is

(31) $$B_{f}\left(p:q\right)=p\log\frac{p}{q}+q-p={\mathrm{kl}}_{+}\left(p:q\right),$$

the *extended scalar Kullback–Leibler divergence*.

We extend the scalar-skew Jensen–Shannon divergence as follows: ${\mathrm{JS}}^{\alpha ,w}\left(p:q\right):={\mathrm{JB}}_{-h}^{\alpha ,\bar{\alpha },w}\left(p:q\right)$ for *h*, the Shannon’s entropy [4] (a strictly concave function [4]).

**Definition** **1** (Weighted vector-skew (α,w)-Jensen–Shannon divergence). *For a vector $\alpha \in {\left[0,1\right]}^{k}$ and a unit positive weight vector $w\in \Delta_{k}$, the (α,w)-Jensen–Shannon divergence between two densities $p,q\in {\bar{P}}_{1}$ is defined by:*

$$\begin{array}{c} {\mathrm{JS}}^{\alpha ,w}\left(p:q\right):=\sum_{i=1}^{k}w_{i}\mathrm{KL}\left({\left(pq\right)}_{\alpha_{i}}:{\left(pq\right)}_{\bar{\alpha }}\right)=h\left({\left(pq\right)}_{\bar{\alpha }}\right)-\sum_{i=1}^{k}w_{i}h\left({\left(pq\right)}_{\alpha_{i}}\right), \end{array}$$

*with $\bar{\alpha }=\sum_{i=1}^{k}w_{i}\alpha_{i}$, where h(p)=−∫p(x)logp(x)dμ(x) denotes the Shannon entropy [4] (i.e., −h is strictly convex).*

This definition generalizes the ordinary JSD; we recover the ordinary Jensen–Shannon divergence when k=2, $\alpha_{1}=0$, $\alpha_{2}=1$, and $w_{1}=w_{2}=\frac{1}{2}$ with $\bar{\alpha }=\frac{1}{2}$: $\mathrm{JS}\left(p,q\right)={\mathrm{JS}}^{\left(0,1\right),\left(\frac{1}{2},\frac{1}{2}\right)}\left(p:q\right)$.

Let ${\mathrm{KL}}_{\alpha ,\beta }\left(p:q\right):=\mathrm{KL}\left({\left(pq\right)}_{\alpha }:{\left(pq\right)}_{\beta }\right)$. Then, we have ${\mathrm{KL}}_{\alpha ,\beta }\left(q:p\right)={\mathrm{KL}}_{1-\alpha ,1-\beta }\left(p:q\right)$. Using this (α,β)-KLD, we have the following identity:

(32) $$\begin{array}{ccc} {\mathrm{JS}}^{\alpha ,w}\left(p:q\right) & = & \sum_{i=1}^{k}w_{i}{\mathrm{KL}}_{\alpha_{i},\bar{\alpha }}\left(p:q\right), \end{array}$$

(33) $$\begin{array}{cc} = & \sum_{i=1}^{k}w_{i}{\mathrm{KL}}_{1-\alpha_{i},1-\bar{\alpha }}\left(q:p\right)={\mathrm{JS}}^{1_{k}-\alpha ,w}\left(q:p\right), \end{array}$$

since $\sum_{i=1}^{k}w_{i}\left(1-\alpha_{i}\right)=\bar{1_{k}-\alpha }=1-\bar{\alpha }$, where $1_{k}=\left(1,\ldots ,1\right)$ is a *k*-dimensional vector of ones.

A very interesting property is that the vector-skew Jensen–Shannon divergences are *f*-divergences [22].

**Theorem** **1.** *The vector-skew Jensen–Shannon divergences ${\mathrm{JS}}^{\alpha ,w}\left(p:q\right)$ are f-divergences for the generator $f_{\alpha ,w}\left(u\right)=\sum_{i=1}^{k}w_{i}\left(\alpha_{i}u+\left(1-\alpha_{i}\right)\right)\log\frac{\left(1-\alpha_{i}\right)+\alpha_{i}u}{\left(1-\bar{\alpha }\right)+\bar{\alpha }u}$ with $\bar{\alpha }=\sum_{i=1}^{k}w_{i}\alpha_{i}$.*

**Proof.**  First, let us observe that the positively weighted sum of *f*-divergences is an *f*-divergence: $\sum_{i=1}^{k}w_{i}I_{f_{i}}\left(p:q\right)=I_{f}\left(p:q\right)$ for the generator $f\left(u\right)=\sum_{i=1}^{k}w_{i}f_{i}\left(u\right)$.

Now, let us express the divergence ${\mathrm{KL}}_{\alpha ,\beta }\left(p:q\right)$ as an *f*-divergence:

(34) $${\mathrm{KL}}_{\alpha ,\beta }\left(p:q\right)=I_{f_{\alpha ,\beta }}\left(p:q\right),$$

with generator

(35) $$f_{\alpha ,\beta }\left(u\right)=\left(\alpha u+1-\alpha \right)\log\frac{(1-\alpha )+\alpha u}{(1-\beta )+\beta u}.$$

Thus, it follows that

(36) $$\begin{array}{ccc} {\mathrm{JS}}^{\alpha ,w}\left(p:q\right) & = & \sum_{i=1}^{k}w_{i}\mathrm{KL}\left({\left(pq\right)}_{\alpha_{i}}:{\left(pq\right)}_{\bar{\alpha }}\right), \end{array}$$

(37) $$\begin{array}{cc} = & \sum_{i=1}^{k}w_{i}I_{f_{\alpha_{i},\bar{\alpha }}}\left(p:q\right), \end{array}$$

(38) $$\begin{array}{cc} = & I_{\sum_{i=1}^{k}w_{i}f_{\alpha_{i},\bar{\alpha }}}\left(p:q\right). \end{array}$$

Therefore, the vector-skew Jensen–Shannon divergence is an *f*-divergence for the following generator:

(39) $$\begin{array}{c} f_{\alpha ,w}\left(u\right)=\sum_{i=1}^{k}w_{i}\left(\alpha_{i}u+\left(1-\alpha_{i}\right)\right)\log\frac{\left(1-\alpha_{i}\right)+\alpha_{i}u}{\left(1-\bar{\alpha }\right)+\bar{\alpha }u}, \end{array}$$

where $\bar{\alpha }=\sum_{i=1}^{k}w_{i}\alpha_{i}$.

When α=(0,1) and $w=(\frac{1}{2},\frac{1}{2})$, we recover the *f*-divergence generator for the JSD:

(40) $$\begin{array}{ccc} f_{\mathrm{JS}}\left(u\right) & = & \frac{1}{2}\log\frac{1}{\frac{1}{2}+\frac{1}{2}u}+\frac{1}{2}u\log\frac{u}{\frac{1}{2}+\frac{1}{2}u}, \end{array}$$

(41) $$\begin{array}{cc} = & \frac{1}{2}\left(\log\frac{2}{1+u}+u\log\frac{2u}{1+u}\right). \end{array}$$

Observe that $f_{\alpha ,w}^{*}\left(u\right)=uf_{\alpha ,w}\left(1/u\right)=f_{1-\alpha ,w}\left(u\right)$, where $1-\alpha :=(1-\alpha_{1},\ldots ,1-\alpha_{k})$.

We also refer the reader to Theorem 4.1 of [31], which defines skew *f*-divergences from any *f*-divergence.  □

**Remark** **1.** *Since the vector-skew Jensen divergence is an f-divergence, we easily obtain Fano and Pinsker inequalities following [32], or reverse Pinsker inequalities following [33,34] (i.e., upper bounds for the vector-skew Jensen divergences using the total variation metric distance), data processing inequalities using [35], etc.*

Next, we show that ${\mathrm{KL}}_{\alpha ,\beta }$ (and ${\mathrm{JS}}^{\alpha ,w}$) are separable convex divergences. Since the *f*-divergences are separable convex, the ${\mathrm{KL}}_{\alpha ,\beta }$ divergences and the ${\mathrm{JS}}^{\alpha ,w}$ divergences are separable convex. For the sake of completeness, we report a simplex explicit proof below.

**Theorem** **2** (Separable convexity). *The divergence ${\mathrm{KL}}_{\alpha ,\beta }\left(p:q\right)$ is strictly separable convex for α≠β and $x\in X_{p}\cap X_{q}$.*

**Proof.**  Let us calculate the second partial derivative of ${\mathrm{KL}}_{\alpha ,\beta }\left(x:y\right)$ with respect to *x*, and show that it is strictly positive:

(42) $$\frac{\partial^{2}}{\partial x^{2}}{\mathrm{KL}}_{\alpha ,\beta }\left(x:y\right)=\frac{{\left(\beta -\alpha \right)}^{2}y^{2}}{{\left(xy\right)}_{\alpha }{\left(xy\right)}_{\beta }^{2}}>0,$$

for x,y>0. Thus, ${\mathrm{KL}}_{\alpha ,\beta }$ is strictly convex on the left argument. Similarly, since ${\mathrm{KL}}_{\alpha ,\beta }\left(y:x\right)={\mathrm{KL}}_{1-\alpha ,1-\beta }\left(x:y\right)$, we deduce that ${\mathrm{KL}}_{\alpha ,\beta }$ is strictly convex on the right argument. Therefore, the divergence ${\mathrm{KL}}_{\alpha ,\beta }$ is separable convex.  □

It follows that the divergence ${\mathrm{JS}}^{\alpha ,w}\left(p:q\right)$ is strictly separable convex, since it is a convex combination of weighted ${\mathrm{KL}}_{\alpha_{i},\bar{\alpha }}$ divergences.

Another way to derive the vector-skew JSD is to decompose the KLD as the difference of the cross-entropy $h^{\times }$ minus the entropy *h* (i.e., KLD is also called the relative entropy):

(43) $$\mathrm{KL}\left(p:q\right)=h^{\times }\left(p:q\right)-h\left(p\right),$$

where $h^{\times }\left(p:q\right):=-\int p\log qd\mu$ and $h\left(p\right):=h^{\times }\left(p:p\right)$ (self cross-entropy). Since $\alpha_{1}h^{\times }\left(p_{1}:q\right)+\alpha_{2}h^{\times }\left(p_{2}:q\right)=h^{\times }\left(\alpha_{1}p_{1}+\alpha_{2}p_{2}:q\right)$ (for $\alpha_{2}=1-\alpha_{1}$), it follows that

(44) $$\begin{array}{ccc} {\mathrm{JS}}^{\alpha ,w}\left(p:q\right) & := & \sum_{i=1}^{k}w_{i}\mathrm{KL}\left({\left(pq\right)}_{\alpha_{i}}:{\left(pq\right)}_{\gamma }\right), \end{array}$$

(45) $$\begin{array}{cc} = & \sum_{i=1}^{k}w_{i}\left(h^{\times }\left({\left(pq\right)}_{\alpha_{i}}:{\left(pq\right)}_{\gamma }\right)-h\left({\left(pq\right)}_{\alpha_{i}}\right)\right), \end{array}$$

(46) $$\begin{array}{cc} = & h^{\times }\left(\sum_{i=1}^{k}w_{i}{\left(pq\right)}_{\alpha_{i}}:{\left(pq\right)}_{\gamma }\right)-\sum_{i=1}^{k}w_{i}h\left({\left(pq\right)}_{\alpha_{i}}\right). \end{array}$$

Here, the “trick” is to choose $\gamma =\bar{\alpha }$ in order to “convert” the cross-entropy into an entropy: $h^{\times }\left(\sum_{i=1}^{k}w_{i}{\left(pq\right)}_{\alpha_{i}}:{\left(pq\right)}_{\gamma }\right)=h\left({\left(pq\right)}_{\bar{\alpha }}\right)$ when $\gamma =\bar{\alpha }$. Then, we end up with

(47) $$\begin{array}{c} {\mathrm{JS}}^{\alpha ,w}\left(p:q\right)=h\left({\left(pq\right)}_{\bar{\alpha }}\right)-\sum_{i=1}^{k}w_{i}h\left({\left(pq\right)}_{\alpha_{i}}\right). \end{array}$$

When $\alpha =(\alpha_{1},\alpha_{2})$ with $\alpha_{1}=0$ and $\alpha_{2}=0$ and $w=\left(w_{1},w_{2}\right)=\left(\frac{1}{2},\frac{1}{2}\right)$, we have $\bar{\alpha }=\frac{1}{2}$, and we recover the Jensen–Shannon divergence:

(48) $$\mathrm{JS}\left(p:q\right)=h\left(\frac{p+q}{2}\right)-\frac{h(p)+h(q)}{2}.$$

Notice that Equation (13) is the usual definition of the Jensen–Shannon divergence, while Equation (48) is the reduced formula of the JSD, which can be interpreted as a Jensen gap for Shannon entropy, hence its name: The *Jensen–Shannon divergence*.

Moreover, if we consider the cross-entropy/entropy extended to positive densities $\tilde{p}$ and $\tilde{q}$:

(49) $$h_{+}^{\times }\left(\tilde{p}:\tilde{q}\right)=-\int \left(\tilde{p}\log\tilde{q}+\tilde{q}\right)d\mu ,\;h_{+}\left(\tilde{p}\right)=h_{+}^{\times }\left(\tilde{p}:\tilde{p}\right)=-\int \left(\tilde{p}\log\tilde{p}+\tilde{p}\right)d\mu ,$$

we get:

(50) $${\mathrm{JS}}_{+}^{\alpha ,w}\left(\tilde{p}:\tilde{q}\right)=\sum_{i=1}^{k}w_{i}{\mathrm{KL}}_{+}\left({\left(\tilde{p}\tilde{q}\right)}_{\alpha_{i}}:{\left(\tilde{p}\tilde{q}\right)}_{\gamma }\right)=h_{+}\left({\left(\tilde{p}\tilde{q}\right)}_{\bar{\alpha }}\right)-\sum_{i=1}^{k}w_{i}h_{+}\left({\left(\tilde{p}\tilde{q}\right)}_{\alpha_{i}}\right).$$

Next, we shall prove that our generalization of the skew Jensen–Shannon divergence to vector-skewing is always bounded. We first start by a lemma bounding the KLD between two mixtures sharing the same components:

**Lemma** **1** (KLD between two *w*-mixtures). *For α∈[0,1] and β∈(0,1), we have:*

$${\mathrm{KL}}_{\alpha ,\beta }\left(p:q\right)=\mathrm{KL}\left({\left(pq\right)}_{\alpha }:{\left(pq\right)}_{\beta }\right)\leq \log\max\left\{\frac{1-\alpha }{1-\beta },\frac{\alpha }{\beta }\right\}.$$

**Proof.**  For p(x),q(x)>0, we have

(51) $$\frac{\left(1-\alpha )p(x)+\alpha q(x\right)}{\left(1-\beta )p(x)+\beta q(x\right)}\leq \max\left\{\frac{1-\alpha }{1-\beta },\frac{\alpha }{\beta }\right\}.$$

Indeed, by considering the two cases α≥β (or equivalently, 1−α≤1−β) and α≤β (or equivalently, 1−α≥1−β), we check that $\left(1-\alpha \right)p\left(x\right)\leq \max\left\{\frac{1-\alpha }{1-\beta },\frac{\alpha }{\beta }\right\}\left(1-\beta \right)p\left(x\right)$ and $\alpha q\left(x\right)\leq \max\left\{\frac{1-\alpha }{1-\beta },\frac{\alpha }{\beta }\right\}\beta q\left(x\right)$. Thus, we have $\frac{\left(1-\alpha )p(x)+\alpha q(x\right)}{\left(1-\beta )p(x)+\beta q(x\right)}\leq \max\left\{\frac{1-\alpha }{1-\beta },\frac{\alpha }{\beta }\right\}$. Therefore, it follows that:

(52) $$\mathrm{KL}\left({\left(pq\right)}_{\alpha }:{\left(pq\right)}_{\beta }\right)\leq \int {\left(pq\right)}_{\alpha }\log\max\left\{\frac{1-\alpha }{1-\beta },\frac{\alpha }{\beta }\right\}d\mu =\log\max\left\{\frac{1-\alpha }{1-\beta },\frac{\alpha }{\beta }\right\}.$$

Notice that we can interpret $\log\max\left\{\frac{1-\alpha }{1-\beta },\frac{\alpha }{\beta }\right\}=\max\left\{\log\frac{1-\alpha }{1-\beta },\log\frac{\alpha }{\beta }\right\}$ as the *∞*-Rényi divergence [36,37] between the following two two-point distributions: (α,1−α) and (β,1−β). See Theorem 6 of [36].

A weaker upper bound is $\mathrm{KL}\left({\left(pq\right)}_{\alpha }:{\left(pq\right)}_{\beta }\right)\leq \log\frac{1}{\beta (1-\beta )}$. Indeed, let us form a partition of the sample space X into two dominance regions:

- $R_{p}:=\left\{x\in X\;:\;q\left(x\right)\leq p\left(x\right)\right\}$ and
- $R_{q}:=\left\{x\in X\;:\;q\left(x\right)>p\left(x\right)\right\}$.

We have ${\left(pq\right)}_{\alpha }\left(x\right)=\left(1-\alpha \right)p\left(x\right)+\alpha q\left(x\right)\leq p\left(x\right)$ for $x\in R_{p}$ and ${\left(pq\right)}_{\alpha }\left(x\right)\leq q\left(x\right)$ for $x\in R_{q}$. It follows that

$$\mathrm{KL}\left({\left(pq\right)}_{\alpha }:{\left(pq\right)}_{\beta }\right)\leq \int_{R_{p}}{\left(pq\right)}_{\alpha }\left(x\right)\log\frac{p(x)}{\left(1-\beta )p(x\right)}d\mu \left(x\right)+\int_{R_{q}}{\left(pq\right)}_{\alpha }\left(x\right)\log\frac{q(x)}{\beta q(x)}d\mu \left(x\right).$$

That is, $\mathrm{KL}\left({\left(pq\right)}_{\alpha }:{\left(pq\right)}_{\beta }\right)\leq -\log\left(1-\beta \right)-\log\beta =\log\frac{1}{\beta (1-\beta )}$. Notice that we allow α∈{0,1} but not β to take the extreme values (i.e., β∈(0,1)).  □

In fact, it is known that for both α,β∈(0,1), $\mathrm{KL}\left({\left(pq\right)}_{\alpha }:{\left(pq\right)}_{\beta }\right)$ amount to compute a Bregman divergence for the Shannon negentropy generator, since $\left\{{\left(pq\right)}_{\gamma }\;:\;\gamma \in \left(0,1\right)\right\}$ defines a *mixture family* [38] of order 1 in information geometry. Hence, it is always finite, as Bregman divergences are always finite (but not necessarily bounded).

By using the fact that

(53) $${\mathrm{JS}}^{\alpha ,w}\left(p:q\right)=\sum_{i=1}^{k}w_{i}\mathrm{KL}\left({\left(pq\right)}_{\alpha_{i}}:{\left(pq\right)}_{\bar{\alpha }}\right),$$

we conclude that the vector-skew Jensen–Shannon divergence is upper-bounded:

**Lemma** **2** (Bounded (w,α)-Jensen–Shannon divergence). *${\mathrm{JS}}^{\alpha ,w}$ is bounded by $\log\frac{1}{\bar{\alpha }\left(1-\bar{\alpha }\right)}$ where $\bar{\alpha }=\sum_{i=1}^{k}w_{i}\alpha_{i}\in \left(0,1\right)$.*

**Proof.**  We have ${\mathrm{JS}}^{\alpha ,w}\left(p:q\right)=\sum_{i}w_{i}\mathrm{KL}\left({\left(pq\right)}_{\alpha_{i}}:{\left(pq\right)}_{\bar{\alpha }}\right)$. Since $0\leq \mathrm{KL}\left({\left(pq\right)}_{\alpha_{i}}:{\left(pq\right)}_{\bar{\alpha }}\right)\leq \log\frac{1}{\bar{\alpha }\left(1-\bar{\alpha }\right)}$, it follows that we have

$$0\leq {\mathrm{JS}}^{\alpha ,w}\left(p:q\right)\leq \log\frac{1}{\bar{\alpha }\left(1-\bar{\alpha }\right)}.$$

Notice that we also have

$${\mathrm{JS}}^{\alpha ,w}\left(p:q\right)\leq \sum_{i}w_{i}\log\max\left\{\frac{1-\alpha_{i}}{1-\bar{\alpha }},\frac{\alpha_{i}}{\bar{\alpha }}\right\}.$$

 □

The vector-skew Jensen–Shannon divergence is symmetric if and only if for each index i∈[k] there exists a matching index σ(i) such that $\alpha_{\sigma (i)}=1-\alpha_{i}$ and $w_{\sigma (i)}=w_{i}$.

For example, we may define the *symmetric scalar α-skew Jensen–Shannon divergence* as

(54) $$\begin{array}{ccc} {\mathrm{JS}}_{s}^{\alpha }\left(p,q\right) & = & \frac{1}{2}\mathrm{KL}\left({\left(pq\right)}_{\alpha }:{\left(pq\right)}_{\frac{1}{2}}\right)+\frac{1}{2}\mathrm{KL}\left({\left(pq\right)}_{1-\alpha }:{\left(pq\right)}_{\frac{1}{2}}\right), \end{array}$$

(55) $$\begin{array}{cc} = & \frac{1}{2}\int {\left(pq\right)}_{\alpha }\log\frac{{\left(pq\right)}_{\alpha }}{{\left(pq\right)}_{\frac{1}{2}}}d\mu +\frac{1}{2}\int {\left(pq\right)}_{1-\alpha }\log\frac{{\left(pq\right)}_{1-\alpha }}{{\left(pq\right)}_{\frac{1}{2}}}d\mu , \end{array}$$

(56) $$\begin{array}{cc} = & \frac{1}{2}\int {\left(qp\right)}_{1-\alpha }\log\frac{{\left(qp\right)}_{1-\alpha }}{{\left(qp\right)}_{\frac{1}{2}}}d\mu ++\frac{1}{2}\int {\left(qp\right)}_{\alpha }\log\frac{{\left(qp\right)}_{\alpha }}{{\left(qp\right)}_{\frac{1}{2}}}d\mu , \end{array}$$

(57) $$\begin{array}{cc} = & h\left({\left(pq\right)}_{\frac{1}{2}}\right)-\frac{h\left({\left(pq\right)}_{\alpha }\right)+h\left({\left(pq\right)}_{1-\alpha }\right)}{2}, \end{array}$$

(58) $$\begin{array}{cc} =: & {\mathrm{JS}}_{s}^{\alpha }\left(q,p\right), \end{array}$$

since it holds that ${\left(ab\right)}_{c}={\left(ba\right)}_{1-c}$ for any a,b,c∈R. Note that ${\mathrm{JS}}_{s}^{\alpha }\left(p,q\right)\neq {\mathrm{JS}}^{\alpha }\left(p,q\right)$.

**Remark** **2.** *We can always symmetrize a vector-skew Jensen–Shannon divergence by doubling the dimension of the skewing vector. Let $\alpha =(\alpha_{1},\cdots ,\alpha_{k})$ and w be the vector parameters of an asymmetric vector-skew JSD, and consider $\alpha^{\prime }=\left(1-\alpha_{1},\ldots ,1-\alpha_{k}\right)$ and w to be the parameters of ${\mathrm{JS}}^{\alpha^{\prime },w}$. Then, ${\mathrm{JS}}^{\left(\alpha ,\alpha^{\prime }\right),\left(\frac{w}{2},\frac{w}{2}\right)}$ is a symmetric skew-vector JSD:*

(59) $$\begin{array}{ccc} {\mathrm{JS}}^{\left(\alpha ,\alpha^{\prime }\right),\left(\frac{w}{2},\frac{w}{2}\right)}\left(p:q\right) & := & \frac{1}{2}{\mathrm{JS}}^{\alpha ,w}\left(p:q\right)+\frac{1}{2}{\mathrm{JS}}^{\alpha^{\prime },w}\left(p:q\right), \end{array}$$

(60) $$\begin{array}{cc} = & \frac{1}{2}{\mathrm{JS}}^{\alpha ,w}\left(p:q\right)+\frac{1}{2}{\mathrm{JS}}^{\alpha ,w}\left(q:p\right)={\mathrm{JS}}^{\left(\alpha ,\alpha^{\prime }\right),\left(\frac{w}{2},\frac{w}{2}\right)}\left(q:p\right). \end{array}$$

Since the vector-skew Jensen–Shannon divergence is an f-divergence for the generator $f_{\alpha ,w}$ (Theorem 1), we can take generator $f_{w,\alpha }^{s}\left(u\right)=\frac{f_{w,\alpha }\left(u\right)+f_{w,\alpha }^{*}\left(u\right)}{2}$ to define the symmetrized f-divergence, where $f_{w,\alpha }^{*}\left(u\right)=uf_{w,\alpha }\left(\frac{1}{u}\right)$ denotes the convex conjugate function. When $f_{\alpha ,w}$ yields a symmetric f-divergence $I_{f_{\alpha ,w}}$, we can apply the generic upper bound of f-divergences (i.e., $I_{f}\leq f\left(0\right)+f^{*}\left(0\right)$) to get the upper bound on the symmetric vector-skew Jensen–Shannon divergences:

(61) $$\begin{array}{ccc} I_{f_{\alpha ,w}}\left(p:q\right) & \leq & f_{\alpha ,w}\left(0\right)+f_{\alpha ,w}^{*}\left(0\right), \end{array}$$

(62) $$\begin{array}{cc} \leq & \sum_{i=1}^{k}w_{i}\left(\left(1-\alpha_{i}\right)\log\frac{1-\alpha_{i}}{1-\bar{\alpha }}+\alpha_{i}\log\frac{\alpha_{i}}{\bar{\alpha }}\right), \end{array}$$

*since*

(63) $$\begin{array}{ccc} f_{\alpha ,w}^{*}\left(u\right) & = & uf_{\alpha ,w}\left(\frac{1}{u}\right), \end{array}$$

(64) $$\begin{array}{cc} = & \sum_{i=1}^{k}w_{i}\left(\left(1-\alpha_{i}\right)u+\alpha_{i}\right)\log\frac{\left(1-\alpha_{i}\right)u+\alpha_{i}}{\left(1-\bar{\alpha }\right)u+\bar{\alpha }}. \end{array}$$

*For example, consider the ordinary Jensen–Shannon divergence with $w=\left(\frac{1}{2},\frac{1}{2}\right)$ and α=(0,1). Then, we find $\mathrm{JS}\left(p:w\right)=I_{f_{\left(0,1\right),\left(\frac{1}{2},\frac{1}{2}\right)}}\left(p:q\right)\leq \frac{1}{2}\log2+\frac{1}{2}\log2=\log2$, the usual upper bound of the JSD.*

As a side note, let us notice that our notation ${\left(pq\right)}_{\alpha }$ allows one to compactly write the following property:

**Property** **1.** *We have $q={\left(qq\right)}_{\lambda }$ for any λ∈[0,1], and ${\left({\left(p_{1}p_{2}\right)}_{\lambda }{\left(q_{1}q_{2}\right)}_{\lambda }\right)}_{\alpha }={\left({\left(p_{1}q_{1}\right)}_{\alpha }{\left(p_{2}q_{2}\right)}_{\alpha }\right)}_{\lambda }$ for any α,λ∈[0,1].*

**Proof.**  Clearly, $q=\left(1-\lambda \right)q+\lambda q=:({\left(qq\right)}_{\lambda })$ for any λ∈[0,1]. Now, we have

(65) $$\begin{array}{ccc} {\left({\left(p_{1}p_{2}\right)}_{\lambda }{\left(q_{1}q_{2}\right)}_{\lambda }\right)}_{\alpha } & = & \left(1-\alpha \right){\left(p_{1}p_{2}\right)}_{\lambda }+\alpha {\left(q_{1}q_{2}\right)}_{\lambda }, \end{array}$$

(66) $$\begin{array}{cc} = & \left(1-\alpha \right)\left(\left(1-\lambda \right)p_{1}+\lambda p_{2}\right)+\alpha \left(\left(1-\lambda \right)q_{1}+\lambda q_{2}\right), \end{array}$$

(67) $$\begin{array}{cc} = & \left(1-\lambda \right)\left(\left(1-\alpha \right)p_{1}+\alpha q_{1}\right)+\lambda \left(\left(1-\alpha \right)p_{2}+\alpha q_{2}\right), \end{array}$$

(68) $$\begin{array}{cc} = & \left(1-\lambda \right){\left(p_{1}q_{1}\right)}_{\alpha }+\lambda {\left(p_{2}q_{2}\right)}_{\alpha }, \end{array}$$

(69) $$\begin{array}{cc} = & {\left({\left(p_{1}q_{1}\right)}_{\alpha }{\left(p_{2}q_{2}\right)}_{\alpha }\right)}_{\lambda }. \end{array}$$

 □

### 2.3. Building Symmetric Families of Vector-Skewed Jensen–Shannon Divergences

We can build infinitely many vector-skew Jensen–Shannon divergences. For example, consider $\alpha =\left(0,1,\frac{1}{3}\right)$ and $w=\left(\frac{1}{3},\frac{1}{3},\frac{1}{3}\right)$. Then, $\bar{\alpha }=\frac{1}{3}+\frac{1}{9}=\frac{4}{9}$, and

(70) $${\mathrm{JS}}^{\alpha ,w}\left(p:q\right)=h\left({\left(pq\right)}_{\frac{4}{9}}\right)-\frac{h\left(p\right)+h\left(q\right)+h\left({\left(pq\right)}_{\frac{1}{3}}\right)}{3}\neq {\mathrm{JS}}^{\alpha ,w}\left(q:p\right).$$

Interestingly, we can also build infinitely many families of *symmetric* vector-skew Jensen–Shannon divergences. For example, consider these two examples that illustrate the construction process:

- Consider k=2. Let (w,1−w) denote the weight vector, and $\alpha =(\alpha_{1},\alpha_{2})$ the skewing vector. We have $\bar{\alpha }=w\alpha_{1}+\left(1-w\right)\alpha_{2}=\alpha_{2}+w\left(\alpha_{1}-\alpha_{2}\right)$. The vector-skew JSD is symmetric iff. $w=1-w=\frac{1}{2}$ (with $\bar{\alpha }=\frac{\alpha_{1}+\alpha_{2}}{2}$) and $\alpha_{2}=1-\alpha_{1}$. In that case, we have $\bar{\alpha }=\frac{1}{2}$, and we obtain the following family of symmetric Jensen–Shannon divergences:
  (71) $$\begin{array}{ccc} {\mathrm{JS}}^{\left(\alpha ,1-\alpha \right),\left(\frac{1}{2},\frac{1}{2}\right)}\left(p,q\right) & = & h\left({\left(pq\right)}_{\frac{1}{2}}\right)-\frac{h\left({\left(pq\right)}_{\alpha }\right)+h\left({\left(pq\right)}_{1-\alpha }\right)}{2}, \end{array}$$
  (72) $$\begin{array}{cc} = & h\left({\left(pq\right)}_{\frac{1}{2}}\right)-\frac{h\left({\left(pq\right)}_{\alpha }\right)+h\left({\left(qp\right)}_{\alpha }\right)}{2}={\mathrm{JS}}^{\left(\alpha ,1-\alpha \right),\left(\frac{1}{2},\frac{1}{2}\right)}\left(q,p\right). \end{array}$$
- Consider k=4, weight vector $w=\left(\frac{1}{3},\frac{1}{3},\frac{1}{6},\frac{1}{6}\right)$, and skewing vector $\alpha =(\alpha_{1},1-\alpha_{1},\alpha_{2},1-\alpha_{2})$ for $\alpha_{1},\alpha_{2}\in \left(0,1\right)$. Then, $\bar{\alpha }=\frac{1}{2}$, and we get the following family of symmetric vector-skew JSDs:
  (73) $$\begin{array}{ccc} {\mathrm{JS}}^{\left(\alpha_{1},\alpha_{2}\right)}\left(p,q\right) & = & h\left({\left(pq\right)}_{\frac{1}{2}}\right)-\frac{2h\left({\left(pq\right)}_{\alpha_{1}}\right)+2h\left({\left(pq\right)}_{1-\alpha_{1}}\right)+h\left({\left(pq\right)}_{\alpha_{2}}\right)+h\left({\left(pq\right)}_{1-\alpha_{2}}\right)}{6}, \end{array}$$
  (74) $$\begin{array}{cc} = & h\left({\left(pq\right)}_{\frac{1}{2}}\right)-\frac{2h\left({\left(pq\right)}_{\alpha_{1}}\right)+2h\left({\left(qp\right)}_{\alpha_{1}}\right)+h\left({\left(pq\right)}_{\alpha_{2}}\right)+h\left({\left(qp\right)}_{\alpha_{2}}\right)}{6}, \end{array}$$
  (75) $$\begin{array}{cc} = & {\mathrm{JS}}^{\left(\alpha_{1},\alpha_{2}\right)}\left(q,p\right). \end{array}$$
- We can similarly carry on the construction of such symmetric JSDs by increasing the dimensionality of the skewing vector.

In fact, we can define

(76) $${\mathrm{JS}}_{s}^{\alpha ,w}\left(p,q\right):=h\left({\left(pq\right)}_{\frac{1}{2}}\right)-\sum_{i=1}^{k}w_{i}\frac{h\left({\left(pq\right)}_{\alpha_{i}}\right)+h\left({\left(pq\right)}_{1-\alpha_{i}}\right)}{2}=\sum_{i=1}^{k}w_{i}{\mathrm{JS}}_{s}^{\alpha_{i}}\left(p,q\right),$$

with

(77) $${\mathrm{JS}}_{s}^{\alpha }\left(p,q\right):=h\left({\left(pq\right)}_{\frac{1}{2}}\right)-\frac{h\left({\left(pq\right)}_{\alpha }\right)+h\left({\left(pq\right)}_{1-\alpha }\right)}{2}.$$

## 3. Jensen–Shannon Centroids on Mixture Families

### 3.1. Mixture Families and Jensen–Shannon Divergences

Consider a mixture family in information geometry [25]. That is, let us give a prescribed set of D+1 linearly independent probability densities $p_{0}\left(x\right),\ldots ,p_{D}\left(x\right)$ defined on the sample space X. A *mixture family* M of order *D* consists of all *strictly* convex combinations of these component densities:

(78) $$M:=\left\{m\left(x;\theta \right):=\sum_{i=1}^{D}\theta^{i}p_{i}\left(x\right)+\left(1-\sum_{i=1}^{D}\theta^{i}\right)p_{0}\left(x\right)\;:\;\theta^{i}>0,\;\sum_{i=1}^{D}\theta^{i}<1\right\}.$$

For example, the family of categorical distributions (sometimes called “multinouilli” distributions) is a mixture family [25]:

(79) $$M=\left\{m_{\theta }\left(x\right)=\sum_{i=1}^{D}\theta_{i}\delta \left(x-x_{i}\right)+\left(1-\sum_{i=1}^{D}\theta_{i}\right)\delta \left(x-x_{0}\right)\right\},$$

where δ(x) is the Dirac distribution (i.e., δ(x)=1 for x=0 and δ(x)=0 for x≠0). Note that the mixture family of categorical distributions can also be interpreted as an exponential family.

Notice that the linearly independent assumption on probability densities is to ensure to have an identifiable model: θ↔m(x;θ).

The KL divergence between two densities of a mixture family M amounts to a Bregman divergence for the Shannon negentropy generator $F\left(\theta \right)=-h\left(m_{\theta }\right)$ (see [38]):

(80) $$\begin{array}{c} \mathrm{KL}\left(m_{\theta_{1}}:m_{\theta_{2}}\right)=B_{F}\left(\theta_{1}:\theta_{2}\right)=B_{-h(m_{\theta })}\left(\theta_{1}:\theta_{2}\right). \end{array}$$

On a mixture manifold M, the mixture density $\left(1-\alpha \right)m_{\theta_{1}}+\alpha m_{\theta_{2}}$ of two mixtures $m_{\theta_{1}}$ and $m_{\theta_{2}}$ of M also belongs to M:

(81) $$\left(1-\alpha \right)m_{\theta_{1}}+\alpha m_{\theta_{2}}=m_{{\left(\theta_{1}\theta_{2}\right)}_{\alpha }}\in M,$$

where we extend the notation ${\left(\theta_{1}\theta_{2}\right)}_{\alpha }:=\left(1-\alpha \right)\theta_{1}+\alpha \theta_{2}$ to vectors $\theta_{1}$ and $\theta_{2}$: ${\left(\theta_{1}\theta_{2}\right)}_{\alpha }^{i}={\left(\theta_{1}^{i}\theta_{2}^{i}\right)}_{\alpha }$.

Thus, the vector-skew JSD amounts to a vector-skew Jensen diversity for the Shannon negentropy convex function $F\left(\theta \right)=-h\left(m_{\theta }\right)$:

(82) $$\begin{array}{ccc} {\mathrm{JS}}^{\alpha ,w}\left(m_{\theta_{1}}:m_{\theta_{2}}\right) & = & \sum_{i=1}^{k}w_{i}\mathrm{KL}\left({\left(m_{\theta_{1}}m_{\theta_{2}}\right)}_{\alpha_{i}}:{\left(m_{\theta_{1}}m_{\theta_{2}}\right)}_{\bar{\alpha }}\right), \end{array}$$

(83) $$\begin{array}{cc} = & \sum_{i=1}^{k}w_{i}\mathrm{KL}\left(m_{{\left(\theta_{1}\theta_{2}\right)}_{\alpha_{i}}}:m_{{\left(\theta_{1}\theta_{2}\right)}_{\bar{\alpha }}}\right), \end{array}$$

(84) $$\begin{array}{cc} = & \sum_{i=1}^{k}w_{i}B_{F}\left({\left(\theta_{1}\theta_{2}\right)}_{\alpha_{i}}:{\left(\theta_{1}\theta_{2}\right)}_{\bar{\alpha }}\right), \end{array}$$

(85) $$\begin{array}{cc} = & {\mathrm{JB}}_{F}^{\alpha ,\bar{\alpha },w}\left(\theta_{1}:\theta_{2}\right), \end{array}$$

(86) $$\begin{array}{cc} = & \sum_{i=1}^{k}w_{i}F\left({\left(\theta_{1}\theta_{2}\right)}_{\alpha_{i}}\right)-F\left({\left(\theta_{1}\theta_{2}\right)}_{\bar{\alpha }}\right), \end{array}$$

(87) $$\begin{array}{cc} = & h\left(m_{{\left(\theta_{1}\theta_{2}\right)}_{\bar{\alpha }}}\right)-\sum_{i=1}^{k}w_{i}h\left(m_{{\left(\theta_{1}\theta_{2}\right)}_{\alpha_{i}}}\right). \end{array}$$

### 3.2. Jensen–Shannon Centroids

Given a set of *n* mixture densities $m_{\theta_{1}},\ldots ,m_{\theta_{n}}$ of M, we seek to calculate the *skew-vector Jensen–Shannon centroid* (or barycenter for non-uniform weights) defined as $m_{\theta^{*}}$, where $\theta^{*}$ is the minimizer of the following objective function (or loss function):

(88) $$L\left(\theta \right):=\sum_{j=1}^{n}\omega_{j}{\mathrm{JS}}^{\alpha ,w}\left(m_{\theta_{k}}:m_{\theta }\right),$$

where $\omega \in \Delta_{n}$ is the weight vector of densities (uniform weight for the centroid and non-uniform weight for a barycenter). This definition of the skew-vector Jensen–Shannon centroid is a generalization of the *Fréchet mean* (the Fréchet mean may not be unique, as it is the case on the sphere for two antipodal points for which their Fréchet means with respect to the geodesic metric distance form a great circle) [39] to non-metric spaces. Since the divergence ${\mathrm{JS}}^{\alpha ,w}$ is strictly separable convex, it follows that the Jensen–Shannon-type centroids are unique when they exist.

Plugging Equation (82) into Equation (88), we get that the calculation of the Jensen–Shannon centroid amounts to the following minimization problem:

(89) $$L\left(\theta \right)=\sum_{j=1}^{n}\omega_{j}\left(\sum_{i=1}^{k}w_{i}F\left({\left(\theta_{j}\theta \right)}_{\alpha_{i}}\right)-F\left({\left(\theta_{j}\theta \right)}_{\bar{\alpha }}\right)\right).$$

This optimization is a *Difference of Convex* (DC) programming optimization, for which we can use the ConCave–Convex procedure [27,40] (CCCP). Indeed, let us define the following two convex functions:

(90) $$\begin{array}{ccc} A(\theta ) & = & \sum_{j=1}^{n}\sum_{i=1}^{k}\omega_{j}w_{i}F\left({\left(\theta_{j}\theta \right)}_{\alpha_{i}}\right), \end{array}$$

(91) $$\begin{array}{ccc} B(\theta ) & = & \sum_{j=1}^{n}\omega_{j}F\left({\left(\theta_{j}\theta \right)}_{\bar{\alpha }}\right). \end{array}$$

Both functions A(θ) and B(θ) are convex since *F* is convex. Then, the minimization problem of Equation (89) to solve can be rewritten as:

(92) $$\min_{\theta }A\left(\theta \right)-B\left(\theta \right).$$

This is a DC programming optimization problem which can be solved iteratively by initializing θ to an arbitrary value $\theta^{\left(0\right)}$ (say, the centroid of the $\theta_{i}$s), and then by updating the parameter at step *t* using the CCCP [27] as follows:

(93) $$\theta^{\left(t+1\right)}={\left(\nabla B\right)}^{-1}\left(\nabla A\left(\theta^{\left(t\right)}\right)\right).$$

Compared to a gradient descent local optimization, there is no required step size (also called “learning” rate) in CCCP.

We have $\nabla A\left(\theta \right)=\sum_{j=1}^{n}\sum_{i=1}^{k}\omega_{j}w_{i}\alpha_{i}\nabla F\left({\left(\theta_{j}\theta \right)}_{\alpha_{i}}\right)$ and $\nabla B\left(\theta \right)=\sum_{j=1}^{n}\omega_{j}\bar{\alpha }\nabla F\left({\left(\theta_{j}\theta \right)}_{\bar{\alpha }}\right)$.

**Figure 1:**
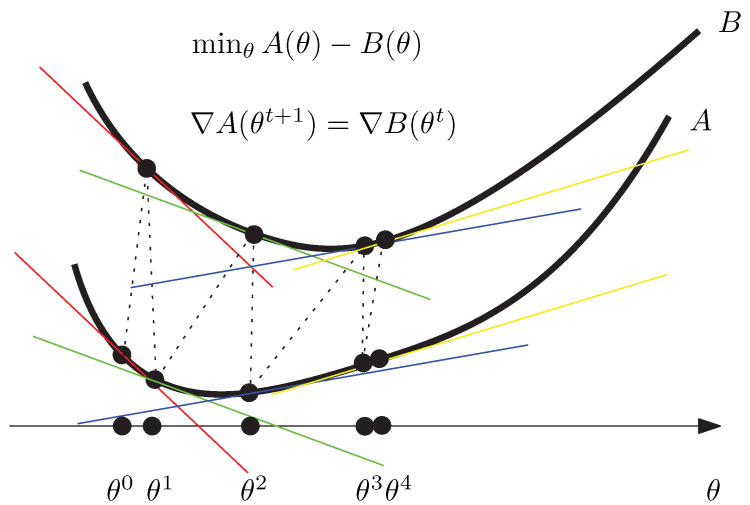
The Convex–ConCave Procedure (CCCP) iteratively updates the parameter θ by aligning the support hyperplanes at θ. In the limit case of convergence to $\theta^{*}$, the support hyperplanes at $\theta^{*}$ are parallel to each other. CCCP finds a local minimum.

The CCCP converges to a local optimum $\theta^{*}$ where the support hyperplanes of the function graphs of *A* and *B* at $\theta^{*}$ are parallel to each other, as depicted in Figure 1. The set of stationary points is {θ:∇A(θ)=∇B(θ)}. In practice, the delicate step is to invert ∇B. Next, we show how to implement this algorithm for the Jensen–Shannon centroid of a set of categorical distributions (i.e., normalized histograms with all non-empty bins).

#### 3.2.1. Jensen–Shannon Centroids of Categorical Distributions

To illustrate the method, let us consider the mixture family of categorical distributions [25]:

(94) $$M=\left\{m_{\theta }\left(x\right)=\sum_{i=1}^{D}\theta_{i}\delta \left(x-x_{i}\right)+\left(1-\sum_{i=1}^{D}\theta_{i}\right)\delta \left(x-x_{0}\right)\right\}.$$

The Shannon negentropy is

(95) $$F\left(\theta \right)=-h\left(m_{\theta }\right)=\sum_{i=1}^{D}\theta_{i}\log\theta_{i}+\left(1-\sum_{i=1}^{D}\theta_{i}\right)\log\left(1-\sum_{i=1}^{D}\theta_{i}\right).$$

We have the partial derivatives

(96) $$\nabla F\left(\theta \right)={\left[\frac{\partial }{\partial \theta_{i}}\right]}_{i},\;\frac{\partial }{\partial \theta_{i}}F\left(\theta \right)=\log\frac{\theta_{i}}{1-\sum_{j=1}^{D}\theta_{j}}.$$

Inverting the gradient ∇F requires us to solve the equation ∇F(θ)=η so that we get $\theta ={\left(\nabla F\right)}^{-1}\left(\eta \right)$. We find that

(97) $$\nabla F^{*}\left(\eta \right)={\left(\nabla F\right)}^{-1}\left(\eta \right)=\frac{1}{1+\sum_{j=1}^{D}\exp\left(\eta_{j}\right)}{\left[\exp\left(\eta_{i}\right)\right]}_{i},\;\theta_{i}={\left(\nabla F^{-1}\left(\eta \right)\right)}_{i}=\frac{\exp(\eta_{i})}{1+\sum_{j=1}^{D}\exp\left(\eta_{j}\right)},\;\forall i\in \left[D\right].$$

Table 1 summarizes the dual view of the family of categorical distributions, either interpreted as an exponential family or as a mixture family.

We have $\mathrm{JS}\left(p_{1},p_{2}\right)=J_{F}\left(\theta_{1},\theta_{2}\right)$ for $p_{1}=m_{\theta_{1}}$ and $p_{2}=m_{\theta_{2}}$, where

(98) $$J_{F}\left(\theta_{1}:\theta_{2}\right)=\frac{F\left(\theta_{1}\right)+F\left(\theta_{2}\right)}{2}-F\left(\frac{\theta_{1}+\theta_{2}}{2}\right),$$

is the Jensen divergence [40]. Thus, to compute the Jensen–Shannon centroid of a set of *n* densities $p_{1},\ldots ,p_{n}$ of a mixture family (with $p_{i}=m_{\theta_{i}}$), we need to solve the following optimization problem for a density $p=m_{\theta }$:

(99) $$\begin{array}{c} \min_{p}\sum_{i}\mathrm{JS}\left(p_{i},p\right), \end{array}$$

(100) $$\begin{array}{c} \min_{\theta }\sum_{i}J_{F}\left(\theta_{i},\theta \right), \end{array}$$

(101) $$\begin{array}{c} \min_{\theta }\sum_{i}\frac{F\left(\theta_{i}\right)+F\left(\theta \right)}{2}-F\left(\frac{\theta_{i}+\theta }{2}\right), \end{array}$$

(102) $$\begin{array}{c} \equiv \min_{\theta }\frac{1}{2}F\left(\theta \right)-\frac{1}{n}\sum_{i}F\left(\frac{\theta_{i}+\theta }{2}\right):=E\left(\theta \right). \end{array}$$

The CCCP algorithm for the Jensen–Shannon centroid proceeds by initializing $\theta^{\left(0\right)}=\frac{1}{n}\sum_{i}\theta_{i}$ (center of mass of the natural parameters), and iteratively updates as follows:

(103) $$\theta^{\left(t+1\right)}={\left(\nabla F\right)}^{-1}\left(\frac{1}{n}\sum_{i}\nabla F\left(\frac{\theta_{i}+\theta^{\left(t\right)}}{2}\right)\right).$$

We iterate until the absolute difference $|E\left(\theta^{\left(t\right)}\right)-E\left(\theta^{\left(t+1\right)}\right)|$ between two successive $\theta^{\left(t\right)}$ and $\theta^{\left(t+1\right)}$ goes below a prescribed threshold value. The convergence of the CCCP algorithm is linear [41] to a local minimum that is a fixed point of the equation

(104) $$\theta =M_{H}\left(\frac{\theta_{1}+\theta }{2},\ldots ,\frac{\theta_{n}+\theta }{2}\right),$$

where $M_{H}\left(v_{1},\ldots ,v_{n}\right):=H^{-1}\left(\sum_{i=1}^{n}H\left(v_{i}\right)\right)$ is a vector generalization of the formula of the quasi-arithmetic means [30,40] obtained for the generator H=∇F. Algorithm 1 summarizes the method for approximating the Jensen–Shannon centroid of a given set of categorical distributions (given a prescribed number of iterations). In the pseudo-code, we used the notation ${}^{\left(t+1\right)}\theta$ instead of $\theta^{\left(t+1\right)}$ in order to highlight the conversion procedures of the natural parameters to/from the mixture weight parameters by using superscript notations for coordinates.

**Algorithm 1:** The CCCP algorithm for computing the Jensen–Shannon centroid of a set of categorical distributions.

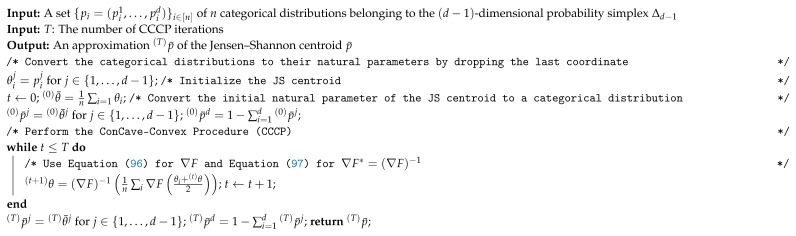



**Figure 2:**
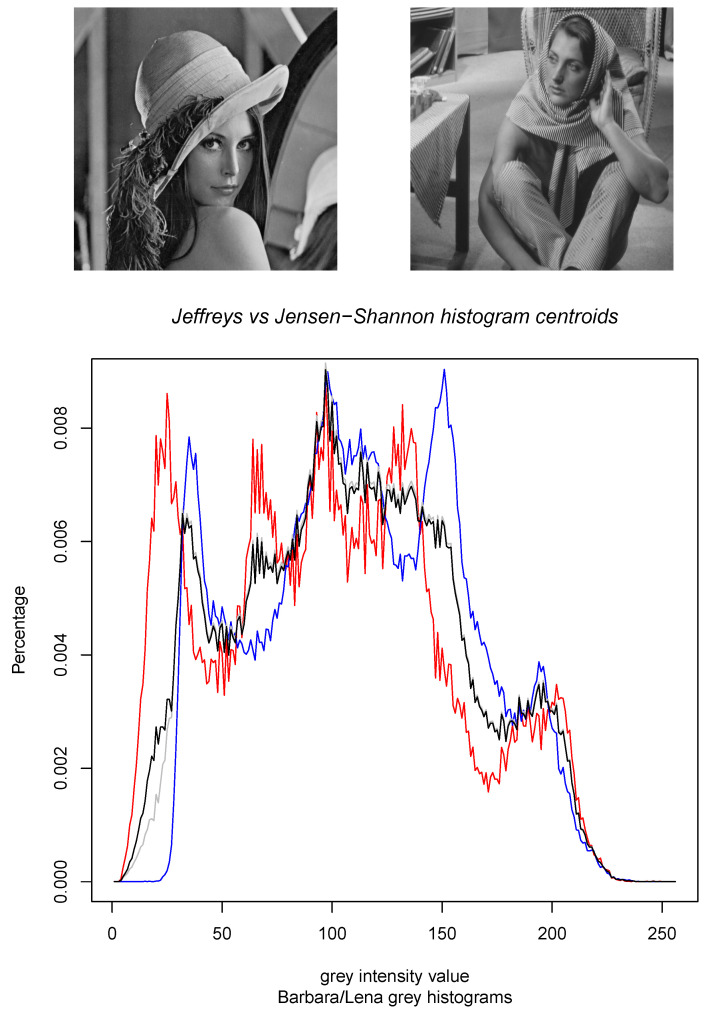
The Jeffreys centroid (grey histogram) and the Jensen–Shannon centroid (black histogram) for two grey normalized histograms of the Lena image (red histogram) and the Barbara image (blue histogram). Although these Jeffreys and Jensen–Shannon centroids look quite similar, observe that there is a major difference between them in the range [0,20] where the blue histogram is zero.

**Figure 3:**
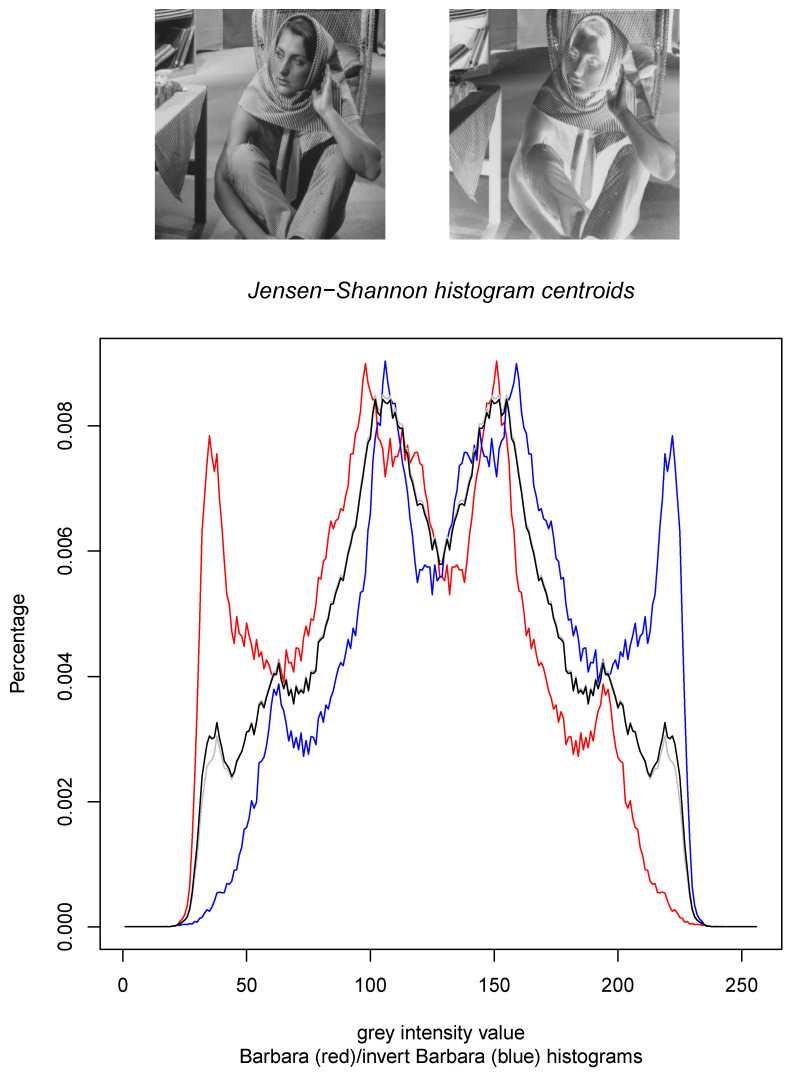
The Jeffreys centroid (grey histogram) and the Jensen–Shannon centroid (black histogram) for the grey normalized histogram of the Barbara image (red histogram) and its negative image (blue histogram which corresponds to the reflection around the vertical axis x=128 of the red histogram).

**Figure 4:**
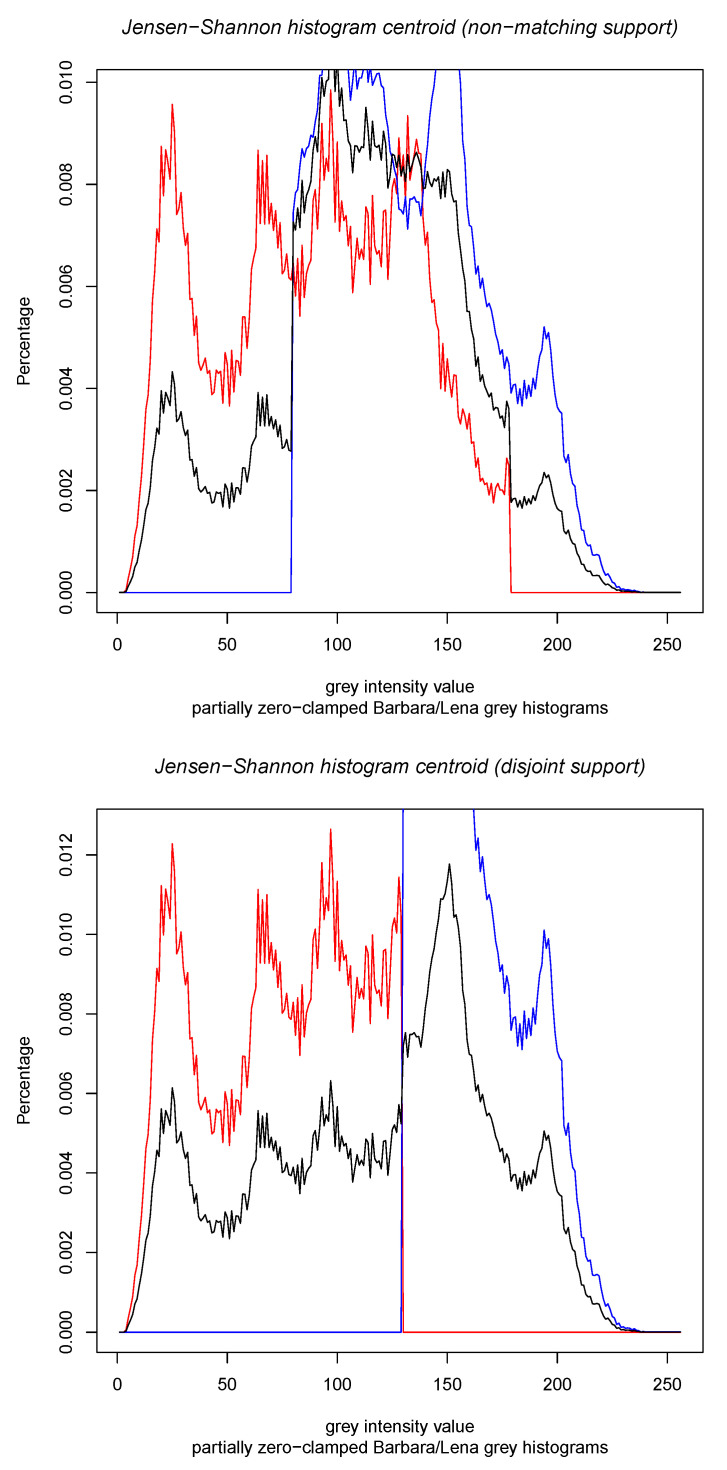
Jensen–Shannon centroid (black histogram) for the clamped grey normalized histogram of the Lena image (red histograms) and the clamped gray normalized histogram of Barbara image (blue histograms). Notice that on the part of the sample space where only one distribution is non-zero, the JS centroid scales that histogram portion.

Figure 2 displays the results of the calculations of the Jeffreys centroid [18] and the Jensen–Shannon centroid for two normalized histograms obtained from grey-valued images of Lena and Barbara. Figure 3 show the Jeffreys centroid and the Jensen–Shannon centroid for the Barbara image and its negative image. Figure 4 demonstrates that the Jensen–Shannon centroid is well defined even if the input histograms do not have coinciding supports. Notice that on the parts of the support where only one distribution is defined, the JS centroid is a scaled copy of that defined distribution.

#### 3.2.2. Special Cases

Let us now consider two special cases:

- For the special case of D=1, the categorical family is the Bernoulli family, and we have F(θ)=θlogθ+(1−θ)log(1−θ) (binary negentropy), $F^{\prime }\left(\theta \right)=\log\frac{\theta }{1-\theta }$ (and $F^{\prime \prime }\left(\theta \right)=\frac{1}{\theta (1-\theta )}>0$) and ${\left(F^{\prime }\right)}^{-1}\left(\eta \right)=\frac{e^{\eta }}{1+e^{\eta }}$. The CCCP update rule to compute the binary Jensen–Shannon centroid becomes
  (105) $$\theta^{\left(t+1\right)}={\left(F^{\prime }\right)}^{-1}\left(\sum_{i}w_{i}F^{\prime }\left(\frac{\theta^{\left(t\right)}+\theta_{i}}{2}\right)\right).$$
- Since the skew-vector Jensen–Shannon divergence formula holds for positive densities:
  (106) $$\begin{array}{ccc} {{\mathrm{JS}}^{+}}^{\alpha ,w}\left(\tilde{p}:\tilde{q}\right) & = & \sum_{i=1}^{k}w_{i}{\mathrm{KL}}^{+}({\left(\tilde{p}\tilde{q}\right)}_{\alpha_{i}}:\left({\left(\tilde{p}\tilde{q}\right)}_{\bar{\alpha }}\right), \end{array}$$
  (107) $$\begin{array}{cc} = & \sum_{i=1}^{k}w_{i}\left(\mathrm{KL}({\left(\tilde{p}\tilde{q}\right)}_{\alpha_{i}}:\left({\left(\tilde{p}\tilde{q}\right)}_{\bar{\alpha }}\right)+\int {\left(\tilde{p}\tilde{q}\right)}_{\bar{\alpha }}d\mu -\underset{=\int {\left(\tilde{p}\tilde{q}\right)}_{\bar{\alpha }}d\mu }{\underset{︸}{\sum_{i=1}^{k}w_{i}\int {\left(\tilde{p}\tilde{q}\right)}_{\alpha_{i}}d\mu }}\right), \end{array}$$
  (108) $$\begin{array}{cc} = & {\mathrm{JS}}^{\alpha ,w}\left(\tilde{p}:\tilde{q}\right), \end{array}$$
  we can *relax* the computation of the Jensen–Shannon centroid by considering 1D separable minimization problems. We then normalize the positive JS centroids to get an approximation of the probability JS centroids. This approach was also considered when dealing with the Jeffreys’ centroid [18]. In 1D, we have F(θ)=θlogθ−θ, $F^{\prime }\left(\theta \right)=\log\theta$ and ${\left(F^{\prime }\right)}^{-1}\left(\eta \right)=e^{\eta }$.

In general, calculating the negentropy for a mixture family with continuous densities sharing the same support is not tractable because of the log-sum term of the differential entropy. However, the following remark emphasizes an extension of the mixture family of categorical distributions:

#### 3.2.3. Some Remarks and Properties

**Remark** **3.** *Consider a mixture family $m\left(\theta \right)=\sum_{i=1}^{D}\theta_{i}p_{i}\left(x\right)+\left(1-\sum_{i=1}^{D}\theta_{i}\right)p_{0}\left(x\right)$ (for a parameter θ belonging to the D-dimensional standard simplex) of probability densities $p_{0}\left(x\right),\ldots ,p_{D}\left(x\right)$ defined respectively on the supports $X_{0},X_{1},\ldots ,X_{D}$. Let $\theta_{0}:=1-\sum_{i=1}^{D}\theta_{i}$. Assume that the support $X_{i}$s of the $p_{i}$s are* mutually non-intersecting*($X_{i}\cap X_{j}=\emptyset$ for all i≠j implying that the D+1 densities are linearly independent) so that $m_{\theta }\left(x\right)=\theta_{i}p_{i}\left(x\right)$ for all $x\in X_{i}$, and let $X=\cup_{i}X_{i}$. Consider Shannon negative entropy $F\left(\theta \right)=-h\left(m_{\theta }\right)$ as a strictly convex function. Then, we have*

(109) $$\begin{array}{ccc} F(\theta ) & = & -h\left(m_{\theta }\right)=\int_{X}m_{\theta }\left(x\right)\log m_{\theta }\left(x\right), \end{array}$$

(110) $$\begin{array}{cc} = & \sum_{i=0}^{D}\theta_{i}\int_{X_{i}}p_{i}\left(x\right)\log\left(\theta_{i}p_{i}\left(x\right)\right)d\mu \left(x\right), \end{array}$$

(111) $$\begin{array}{cc} = & \sum_{i=0}^{D}\theta_{i}\log\theta_{i}-\sum_{i=0}^{D}\theta_{i}h\left(p_{i}\right). \end{array}$$

*Note that the term $\sum_{i}\theta_{i}h\left(p_{i}\right)$ is affine in θ, and Bregman divergences are defined up to affine terms so that the Bregman generator F is equivalent to the Bregman generator of the family of categorical distributions. This example generalizes the ordinary mixture family of categorical distributions where the $p_{i}$s are distinct Dirac distributions. Note that when the support of the component distributions are not pairwise disjoint, the (neg)entropy may not be analytic [42] (e.g., mixture of the convex weighting of two prescribed distinct Gaussian distributions). This contrasts with the fact that the cumulant function of an exponential family is always real-analytic [43]. Observe that the term $\sum_{i}\theta_{i}h\left(p_{i}\right)$ can be interpreted as a conditional entropy: $\sum_{i}\theta_{i}h\left(p_{i}\right)=h\left(X|\Theta \right)$ where $\mathrm{Pr}\left(\Theta =i\right)=\theta_{i}$ and $\mathrm{Pr}\left(X\in S|\Theta =i\right)=\int_{S}p_{i}\left(x\right)d\mu \left(x\right)$.*

Notice that we can truncate an exponential family [25] to get a (potentially non-regular [44]) exponential family for defining the $p_{i}$s on mutually non-intersecting domains $X_{i}$s. The entropy of a natural exponential family $\left\{e\left(x:\theta \right)=\exp\left(x^{⊤}\theta -\psi \left(\theta \right)\right)\;\;:\;\;\theta \in \Theta \right\}$ with cumulant function ψ(θ) and natural parameter space Θ is $-\psi^{*}\left(\eta \right)$, where η=∇ψ(θ), and $\psi^{*}$ is the Legendre convex conjugate [45]: $h\left(e\left(x:\theta \right)\right)=-\psi^{*}\left(\nabla \psi \left(\theta \right)\right)$.

In general, the entropy and cross-entropy between densities of a mixture family (whether the distributions have disjoint supports or not) can be calculated in closed-form.

**Property** **2.** The entropy of a density belonging to a mixture family M is $h\left(m_{\theta }\right)=-F\left(\theta \right)$, and the cross-entropy between two mixture densities $m_{\theta_{1}}$ and $m_{\theta_{2}}$ is $h^{\times }\left(m_{\theta_{1}}:m_{\theta_{2}}\right)=-F\left(\theta_{2}\right)-{\left(\theta_{1}-\theta_{2}\right)}^{⊤}\eta_{2}=F^{*}\left(\eta_{2}\right)-\theta_{1}^{⊤}\eta_{2}$.

**Proof.**  Let us write the KLD as the difference between the cross-entropy minus the entropy [4]:

(112) $$\begin{array}{ccc} \mathrm{KL}(m_{\theta_{1}}:m_{\theta_{2}}) & = & h^{\times }\left(m_{\theta_{1}}:m_{\theta_{2}}\right)-h\left(m_{\theta_{1}}\right), \end{array}$$

(113) $$\begin{array}{cc} = & B_{F}\left(\theta_{1}:\theta_{2}\right), \end{array}$$

(114) $$\begin{array}{cc} = & F\left(\theta_{1}\right)-F\left(\theta_{2}\right)-{\left(\theta_{1}-\theta_{2}\right)}^{⊤}\nabla F\left(\theta_{2}\right). \end{array}$$

Following [45], we deduce that $h\left(m_{\theta }\right)=-F\left(\theta \right)+c$ and $h^{\times }\left(m_{\theta_{1}}:m_{\theta_{2}}\right)=-F\left(\theta_{2}\right)-{\left(\theta_{1}-\theta_{2}\right)}^{⊤}\eta_{2}-c$ for a constant *c*. Since $F\left(\theta \right)=-h\left(m_{\theta }\right)$ by definition, it follows that c=0 and that $h^{\times }\left(m_{\theta_{1}}:m_{\theta_{2}}\right)=-F\left(\theta_{2}\right)-{\left(\theta_{1}-\theta_{2}\right)}^{⊤}\eta_{2}=F^{*}\left(\eta_{2}\right)-\theta_{1}^{⊤}\eta_{2}$ where η=∇F(θ).  □

Thus, we can numerically compute the Jensen–Shannon centroids (or barycenters) of a set of densities belonging to a mixture family. This includes the case of categorical distributions and the case of Gaussian Mixture Models (GMMs) with prescribed Gaussian components [38] (although in this case, the negentropy needs to be stochastically approximated using Monte Carlo techniques [46]). When the densities do not belong to a mixture family (say, the Gaussian family, which is an exponential family [25]), we face the problem that the mixture of two densities does not belong to the family anymore. One way to tackle this problem is to project the mixture onto the Gaussian family. This corresponds to an *m*-projection (mixture projection) which can be interpreted as a Maximum Entropy projection of the mixture [25,47]).

Notice that we can perform fast *k*-means clustering without centroid calculations using a generalization of the *k*-means++ probabilistic initialization [48,49]. See [50] for details of the generalized *k*-means++ probabilistic initialization defined according to an arbitrary divergence.

Finally, let us notice some decompositions of the Jensen–Shannon divergence and the skew Jensen divergences.

**Remark** **4.** *We have the following decomposition for the Jensen–Shannon divergence:*

(115) $$\begin{array}{ccc} \mathrm{JS}(p_{1},p_{2}) & = & h\left(\frac{p_{1}+p_{2}}{2}\right)-\frac{h\left(p_{1}\right)+h\left(p_{2}\right)}{2}, \end{array}$$

(116) $$\begin{array}{cc} = & h_{\mathrm{JS}}^{\times }\left(p_{1}:p_{2}\right)-h_{\mathrm{JS}}\left(p_{2}\right)\geq 0, \end{array}$$

*where*

(117) $$h_{\mathrm{JS}}^{\times }\left(p_{1}:p_{2}\right)=h\left(\frac{p_{1}+p_{2}}{2}\right)-\frac{1}{2}h\left(p_{1}\right),$$

*and $h_{\mathrm{JS}}\left(p_{2}\right)=h_{\mathrm{JS}}^{\times }\left(p_{2}:p_{2}\right)=h\left(p_{2}\right)-\frac{1}{2}h\left(p_{2}\right)=\frac{1}{2}h\left(p_{2}\right)$. This decomposition bears some similarity with the KLD decomposition viewed as the cross-entropy minus the entropy (with the cross-entropy always upper-bounding the entropy).*

*Similarly, the α-skew Jensen divergence*

(118) $$J_{F}^{\alpha }\left(\theta_{1}:\theta_{2}\right):={\left(F\left(\theta_{1}\right)F\left(\theta_{2}\right)\right)}_{\alpha }-F\left({\left(\theta_{1}\theta_{2}\right)}_{\alpha }\right),\;\alpha \in \left(0,1\right)$$

*can be decomposed as the sum of the information $I_{F}^{\alpha }\left(\theta_{1}\right)=\left(1-\alpha \right)F\left(\theta_{1}\right)$ minus the cross-information $C_{F}^{\alpha }\left(\theta_{1}:\theta_{2}\right):=F\left({\left(\theta_{1}\theta_{2}\right)}_{\alpha }\right)-\alpha F\left(\theta_{2}\right)$:*

(119) $$J_{F}^{\alpha }\left(\theta_{1}:\theta_{2}\right)=I_{F}^{\alpha }\left(\theta_{1}\right)-C_{F}^{\alpha }\left(\theta_{1}:\theta_{2}\right)\geq 0.$$

*Notice that the information $I_{F}^{\alpha }\left(\theta_{1}\right)$ is the self cross-information: $I_{F}^{\alpha }\left(\theta_{1}\right)=C_{F}^{\alpha }\left(\theta_{1}:\theta_{1}\right)=\left(1-\alpha \right)F\left(\theta_{1}\right)$. Recall that the convex information is the negentropy where the entropy is concave. For the Jensen–Shannon divergence on the mixture family of categorical distributions, the convex generator $F\left(\theta \right)=-h\left(m_{\theta }\right)=\sum_{i=1}^{D}\theta^{i}\log\theta^{i}$ is the Shannon negentropy.*

Finally, let us briefly mention the *Jensen–Shannon diversity* [30] which extends the Jensen–Shannon divergence to a weighted set of densities as follows:

(120) $$\mathrm{JS}\left(p_{1},\ldots ,p_{k};w_{1},\ldots ,w_{k}\right):=\sum_{i=1}^{k}w_{i}\mathrm{KL}\left(p_{i}:\bar{p}\right),$$

where $\bar{p}=\sum_{i=1}^{k}w_{i}p_{i}$. The Jensen–Shannon diversity plays the role of the variance of a cluster with respect to the KLD. Indeed, let us state the compensation identity [51]: For any *q*, we have

(121) $$\sum_{i=1}^{k}w_{i}\mathrm{KL}\left(p_{i}:q\right)=\sum_{i=1}^{k}w_{i}\mathrm{KL}\left(p_{i}:\bar{p}\right)+\mathrm{KL}\left(\bar{p}:q\right).$$

Thus, the cluster center defined as the minimizer of $\sum_{i=1}^{k}w_{i}\mathrm{KL}\left(p_{i}:q\right)$ is the centroid $\bar{p}$, and

(122) $$\sum_{i=1}^{k}w_{i}\mathrm{KL}\left(p_{i}:\bar{p}\right)=\mathrm{JS}\left(p_{1},\ldots ,p_{k};w_{1},\ldots ,w_{k}\right).$$

## 4. Conclusions and Discussion

The Jensen–Shannon divergence [6] is a renown symmetrization of the Kullback–Leibler oriented divergence that enjoys the following three essential properties:

1. It is always bounded,
2. it applies to densities with potentially different supports, and
3. it extends to unnormalized densities while enjoying the same formula expression.

This JSD plays an important role in machine learning and in deep learning for studying Generative Adversarial Networks (GANs) [52]. Traditionally, the JSD has been skewed with a scalar parameter [19,53] α∈(0,1). In practice, it has been experimentally demonstrated that skewing divergences may significantly improve the performance of some tasks (e.g., [21,54]).

In general, we can symmetrize the KLD KL(p:q) by taking an *abstract mean* (we require a symmetric mean M(x,y)=M(y,x) with the in-betweenness property: min{x,y}≤M(x,y)≤max{x,y}) *M* between the two orientations KL(p:q) and KL(q:p):

(123) $${\mathrm{KL}}_{M}\left(p,q\right):=M\left(\mathrm{KL}\left(p:q\right),\mathrm{KL}\left(q:p\right)\right).$$

We recover the Jeffreys divergence by taking the arithmetic mean twice (i.e., J(p,q)=2A(KL(p:q),KL(q:p)) where $A\left(x,y\right)=\frac{x+y}{2}$), and the resistor average divergence [55] by taking the harmonic mean (i.e., $R_{\mathrm{KL}}\left(p,q\right)=H\left(\mathrm{KL}\left(p:q\right),\mathrm{KL}\left(q:p\right)\right)=\frac{2\mathrm{KL}(p:q)\mathrm{KL}(q:p)}{\mathrm{KL}(p:q)+\mathrm{KL}(q:p)}$ where $H\left(x,y\right)=\frac{2}{\frac{1}{x}+\frac{1}{y}}$). When we take the limit of Hölder power means, we get the following extremal symmetrizations of the KLD:

(124) $$\begin{array}{ccc} {\mathrm{KL}}^{\min}\left(p:q\right) & = & \min\left\{\mathrm{KL}\left(p:q\right),\mathrm{KL}\left(q:p\right)\right\}={\mathrm{KL}}^{\min}\left(q:p\right), \end{array}$$

(125) $$\begin{array}{ccc} {\mathrm{KL}}^{\max}\left(p:q\right) & = & \max\left\{\mathrm{KL}\left(p:q\right),\mathrm{KL}\left(q:p\right)\right\}={\mathrm{KL}}^{\max}\left(q:p\right). \end{array}$$

In this work, we showed how to *vector-skew* the JSD while preserving the above three properties. These new families of *weighted vector-skew Jensen–Shannon divergences* may allow one to fine-tune the dissimilarity in applications by replacing the skewing scalar parameter of the JSD by a vector parameter (informally, adding some “knobs” for tuning a divergence). We then considered computing the Jensen–Shannon centroids of a set of densities belonging to a mixture family [25] by using the convex–concave procedure [27].

In general, we can vector-skew any arbitrary divergence *D* by using two *k*-dimensional vectors $\alpha \in {\left[0,1\right]}^{k}$ and $\beta \in {\left[0,1\right]}^{k}$ (with α≠β) by building a weighted separable divergence as follows:

(126) $$D^{\alpha ,\beta ,w}\left(p:q\right):=\sum_{i=1}^{k}w_{i}D\left({\left(pq\right)}_{\alpha_{i}}:{\left(pq\right)}_{\beta_{i}}\right)=D^{1_{k}-\alpha ,1_{k}-\beta ,w}\left(q:p\right),\;\alpha \neq \beta .$$

This bi-vector-skew divergence unifies the Jeffreys divergence with the Jensen–Shannon α-skew divergence by setting the following parameters:

(127) $$\begin{array}{ccc} {\mathrm{KL}}^{\left(0,1),(1,0),(1,1\right)}\left(p:q\right) & = & \mathrm{KL}(p:q)+\mathrm{KL}(q:p)=J(p,q), \end{array}$$

(128) $$\begin{array}{ccc} {\mathrm{KL}}^{\left(0,\alpha \right),\left(1,1-\alpha \right),\left(\frac{1}{2},\frac{1}{2}\right)}\left(p:q\right) & = & \frac{1}{2}\mathrm{KL}\left(p:{\left(pq\right)}_{\alpha }\right)+\frac{1}{2}\mathrm{KL}\left(q:{\left(pq\right)}_{\alpha }\right). \end{array}$$

We have shown in this paper that interesting properties may occur when the skewing vector β is purposely correlated to the skewing vector α: Namely, for the bi-vector-skew Bregman divergences with $\beta =(\bar{\alpha },\ldots ,\bar{\alpha })$ and $\bar{\alpha }=\sum_{i}w_{i}\alpha_{i}$, we obtain an equivalent Jensen diversity for the Jensen–Bregman divergence, and, as a byproduct, a vector-skew generalization of the Jensen–Shannon divergence.

## Figures and Tables

**Table 1:** Two views of the family of categorical distributions with *d* choices: An exponential family or a mixture family of order D=d−1. Note that the Bregman divergence associated to the exponential family view corresponds to the reverse Kullback–Leibler (KL) divergence, while the Bregman divergence associated to the mixture family view corresponds to the KL divergence.

	Exponential Family	Mixture Family
pdf	$p_{\theta }\left(x\right)=\prod_{i=1}^{d}p_{i}^{t_{i}\left(x\right)},p_{i}=\mathrm{Pr}\left(x=e_{i}\right),t_{i}\left(x\right)\in \left\{0,1\right\},\sum_{i=1}^{d}t_{i}\left(x\right)=1$	$m_{\theta }\left(x\right)=\sum_{i=1}^{d}p_{i}\delta_{e_{i}}\left(x\right)$
primal θ	$\theta_{i}=\log\frac{p_{i}}{p_{d}}$	$\theta_{i}=p_{i}$
F(θ)	$\log(1+\sum_{i=1}^{D}\exp\left(\theta_{i}\right))$	$\theta_{i}\log\theta_{i}+\left(1-\sum_{i=1}^{D}\theta_{i}\right)\log\left(1-\sum_{i=1}^{D}\theta_{i}\right)$
dual η=∇F(θ)	$\frac{e^{\theta_{i}}}{1+\sum_{j=1}^{D}\exp\left(\theta_{j}\right)}$	$\log\frac{\theta_{i}}{1-\sum_{j=1}^{D}\theta_{j}}$
primal $\theta =\nabla F^{*}\left(\eta \right)$	$\log\frac{\eta_{i}}{1-\sum_{j=1}^{D}\eta_{j}}$	$\frac{e^{\theta_{i}}}{1+\sum_{j=1}^{D}\exp\left(\theta_{j}\right)}$
$F^{*}\left(\eta \right)$	$\sum_{i=1}^{D}\eta_{i}\log\eta_{i}+\left(1-\sum_{j=1}^{D}\eta_{j}\right)\log\left(1-\sum_{j=1}^{D}\eta_{j}\right)$	$\log(1+\sum_{i=1}^{D}\exp\left(\eta_{i}\right))$
Bregman divergence	$B_{F}\left(\theta :\theta^{\prime }\right)={\mathrm{KL}}^{*}\left(p_{\theta }:p_{\theta^{\prime }}\right)$	$B_{F}\left(\theta :\theta^{\prime }\right)=\mathrm{KL}\left(m_{\theta }:m_{\theta^{\prime }}\right)$
	$=\mathrm{KL}(p_{\theta^{\prime }}:p_{\theta })$	

## References

[1] Billingsley P. (2008). Probability and Measure.

[2] Deza M.M., Deza E. (2009). Encyclopedia of Distances.

[3] Basseville M. (2013). Divergence measures for statistical data processing—An annotated bibliography. Signal Process..

[4] Cover T.M., Thomas J.A. (2012). Elements of Information Theory.

[5] Nielsen F. (2019). On the Jensen–Shannon Symmetrization of Distances Relying on Abstract Means. Entropy.

[6] Lin J. (1991). Divergence measures based on the Shannon entropy. IEEE Trans. Inf. Theory.

[7] Sason I. Tight bounds for symmetric divergence measures and a new inequality relating *f*-divergences. Proceedings of the 2015 IEEE Information Theory Workshop (ITW).

[8] Wong A.K., You M. (1985). Entropy and distance of random graphs with application to structural pattern recognition. IEEE Trans. Pattern Anal. Mach. Intell..

[9] Endres D.M., Schindelin J.E. (2003). A new metric for probability distributions. IEEE Trans. Inf. Theory.

[10] Kafka P., Österreicher F., Vincze I. (1991). On powers of *f*-divergences defining a distance. Stud. Sci. Math. Hung..

[11] Fuglede B. (2005). Spirals in Hilbert space: With an application in information theory. Expo. Math..

[12] Acharyya S., Banerjee A., Boley D. Bregman divergences and triangle inequality. Proceedings of the 2013 SIAM International Conference on Data Mining.

[13] Naghshvar M., Javidi T., Wigger M. (2015). Extrinsic Jensen–Shannon divergence: Applications to variable-length coding. IEEE Trans. Inf. Theory.

[14] Bigi B. (2003). Using Kullback-Leibler distance for text categorization. European Conference on Information Retrieval.

[15] Chatzisavvas K.C., Moustakidis C.C., Panos C. (2005). Information entropy, information distances, and complexity in atoms. J. Chem. Phys..

[16] Yurdakul B. (2018). Statistical Properties of Population Stability Index. Ph.D. Thesis.

[17] Jeffreys H. (1946). An invariant form for the prior probability in estimation problems. Proc. R. Soc. Lond. A.

[18] Nielsen F. (2013). Jeffreys centroids: A closed-form expression for positive histograms and a guaranteed tight approximation for frequency histograms. IEEE Signal Process. Lett..

[19] Lee L. (1999). Measures of Distributional Similarity. Proceedings of the 37th Annual Meeting of the Association for Computational Linguistics on Computational Linguistics, ACL ’99.

[20] Nielsen F. (2010). A family of statistical symmetric divergences based on Jensen’s inequality. arXiv.

[21] Lee L. On the effectiveness of the skew divergence for statistical language analysis. Proceedings of the 8th International Workshop on Artificial Intelligence and Statistics (AISTATS 2001).

[22] Csiszár I. (1967). Information-type measures of difference of probability distributions and indirect observation. Stud. Sci. Math. Hung..

[23] Ali S.M., Silvey S.D. (1966). A general class of coefficients of divergence of one distribution from another. J. R. Stat. Soc. Ser. B (Methodol.).

[24] Sason I. (2018). On *f*-divergences: Integral representations, local behavior, and inequalities. Entropy.

[25] Amari S.I. (2016). Information Geometry and Its Applications.

[26] Jiao J., Courtade T.A., No A., Venkat K., Weissman T. (2014). Information measures: The curious case of the binary alphabet. IEEE Trans. Inf. Theory.

[27] Yuille A.L., Rangarajan A. The concave-convex procedure (CCCP). Proceedings of the Neural Information Processing Systems 2002.

[28] Nielsen F., Nock R. (2011). Skew Jensen-Bregman Voronoi diagrams. Transactions on Computational Science XIV.

[29] Banerjee A., Merugu S., Dhillon I.S., Ghosh J. (2005). Clustering with Bregman divergences. J. Mach. Learn. Res..

[30] Nielsen F., Nock R. (2009). Sided and symmetrized Bregman centroids. IEEE Trans. Inf. Theory.

[31] Melbourne J., Talukdar S., Bhaban S., Madiman M., Salapaka M.V. On the Entropy of Mixture distributions. http://box5779.temp.domains/~jamesmel/publications/.

[32] Guntuboyina A. (2011). Lower bounds for the minimax risk using *f*-divergences, and applications. IEEE Trans. Inf. Theory.

[33] Sason I., Verdu S. (2016). *f*-divergence Inequalities. IEEE Trans. Inf. Theory.

[34] Melbourne J., Madiman M., Salapaka M.V. Relationships between certain *f*-divergences. Proceedings of the 57th Annual Allerton Conference on Communication, Control, and Computing (Allerton).

[35] Sason I. (2019). On Data-Processing and Majorization Inequalities for *f*-Divergences with Applications. Entropy.

[36] Van Erven T., Harremos P. (2014). Rényi divergence and Kullback-Leibler divergence. IEEE Trans. Inf. Theory.

[37] Xu P., Melbourne J., Madiman M. Infinity-Rényi entropy power inequalities. Proceedings of the 2017 IEEE International Symposium on Information Theory (ISIT).

[38] Nielsen F., Nock R. On the geometry of mixtures of prescribed distributions. Proceedings of the 2018 IEEE International Conference on Acoustics, Speech and Signal Processing (ICASSP).

[39] Fréchet M. (1948). Les éléments aléatoires de nature quelconque dans un espace distancié. Ann. De L’institut Henri PoincarÉ.

[40] Nielsen F., Boltz S. (2011). The Burbea-Rao and Bhattacharyya centroids. IEEE Trans. Inf. Theory.

[41] Lanckriet G.R., Sriperumbudur B.K. On the convergence of the concave-convex procedure. Proceedings of the Advances in Neural Information Processing Systems 22 (NIPS 2009).

[42] Nielsen F., Sun K. (2016). Guaranteed bounds on information-theoretic measures of univariate mixtures using piecewise log-sum-exp inequalities. Entropy.

[43] Springer Verlag GmbH, European Mathematical Society Encyclopedia of Mathematics. https://www.encyclopediaofmath.org/.

[44] Del Castillo J. (1994). The singly truncated normal distribution: A non-steep exponential family. Ann. Inst. Stat. Math..

[45] Nielsen F., Nock R. Entropies and cross-entropies of exponential families. Proceedings of the 2010 IEEE International Conference on Image Processing.

[46] Nielsen F., Hadjeres G. (2018). Monte Carlo information geometry: The dually flat case. arXiv.

[47] Schwander O., Nielsen F. (2013). Learning mixtures by simplifying kernel density estimators. Matrix Information Geometry.

[48] Arthur D., Vassilvitskii S. *k*-means++: The advantages of careful seeding. Proceedings of the Eighteenth Annual ACM-SIAM Symposium on Discrete Algorithms (SODA’07).

[49] Nielsen F., Nock R., Amari S.I. (2014). On clustering histograms with *k*-means by using mixed *α*-divergences. Entropy.

[50] Nielsen F., Nock R. Total Jensen divergences: Definition, properties and clustering. Proceedings of the 2015 IEEE International Conference on Acoustics, Speech and Signal Processing (ICASSP).

[51] Topsøe F. (2001). Basic concepts, identities and inequalities-the toolkit of information theory. Entropy.

[52] Goodfellow I., Pouget-Abadie J., Mirza M., Xu B., Warde-Farley D., Ozair S., Courville A., Bengio Y. Generative adversarial nets. Proceedings of the Advances in Neural Information Processing Systems 27 (NIPS 2014).

[53] Yamano T. (2019). Some bounds for skewed *α*-Jensen-Shannon divergence. Results Appl. Math..

[54] Kotlerman L., Dagan I., Szpektor I., Zhitomirsky-Geffet M. (2010). Directional distributional similarity for lexical inference. Nat. Lang. Eng..

[55] Johnson D., Sinanovic S. (2001). Symmetrizing the Kullback-Leibler distance. IEEE Trans. Inf. Theory.

